# Supplementary material for: Interleukin-21 expressing oncolytic vaccinia virus elicits systemic anti-tumor immune response and synergizes with immunotherapies
Source: Mol Ther Oncol. 2026 Sep 18;34(4):201348. doi: 10.1016/j.omton.2026.201348 (PMC13720255; doi:10.1016/j.omton.2026.201348)
Supplement: Supplementary file 1 [file mmc1.pdf]

OMTON, Volume *34*

Supplemental information

Interleukin-21 expressing oncolytic vaccinia virus elicits systemic anti-tumor immune response and synergizes with immunotherapies

Rong Zhang, Tingting Wang, Yu Wang, Chenggang Hong, Yun Qin, Kangchen Ye, Hu Zhao, Fang Hu, Jinlu Cai, and Jin Fu

Supplemental Methods

Cells. A375, MDA-MB-231, and U251 were obtained from the Cell Bank of the Chinese Academy of Sciences. Other cell lines, including MNNG/HOS C1, HCT-116, MCF-7, PANC1 and SKOV3 were purchased from the American Type Culture Collection. All cells, excluding MDA-MB-231 cells, were maintained in their respective recommended culture media supplemented with 10% FBS, 100 U/mL penicillin, and 100 μg/mL streptomycin, and incubated at 37°C in a humidified atmosphere containing 5% CO2. MDA-MB-231 cells were cultured at 37°C in the absent of CO2.

Cell cytotoxicity. To assess the oncolytic activity of hV01, we performed a dose-response analysis using eight human cancer cell lines: MNNG/HOS C1, U251, SKOV3, A375, MCF-7, PANC-1, MDA-MB-231, and HCT-116. Cells were seeded at a density of 5,000 cells per well in 96-well plates and infected with serial dilutions of hV01. After 48 hpi, cell viability was quantified using the MTT assay (Invitrogen, Shanghai, China) according to the manufacturer's instructions. Absorbance was measured at 490 nm using a VICTOR Nivo™ Multimode Plate Reader (PerkinElmer). Oncolytic efficacy was expressed as the percentage of cell viability relative to mock-infected controls. Dose-response curves were generated to determine the half-maximal inhibitory concentration (IC_{50}) of hV01.

To evaluate hV01-induced ICD, MDA-MB-231 cells were seeded at 5×10^4 cells/well in 24-well plates and infected with hV01 or a mock control virus at MOI of 1.0. After 2 hpi, the culture medium was replaced with fresh complete medium containing effector cells at an E:T ratio of 5:1 for hPBMCs and 0.5:1 for hNK cells. The effector groups included: hNK cells, hNK cells pre-activated with hrIL-21, hPBMCs, or hPBMCs pre-activated with hrIL-21. Following 48 hours of co-culture, cells were harvested and stained with Fixable Viability Dye eFluor™ 780

(ThermoFisher Scientific). Cell viability was quantified using a NovoCyte flow cytometer (Agilent Technologies, Shanghai, China).

Animal studies. Syngeneic C57BL/6N mice were s.c. inoculated with 5×10^5 MC38 cells in the right flank. Treatment consisted of a single i.t. injection of mV01 (3×10^4 PFU) on day 0, followed by i.v. administration of mPD-1 antibody (5 mg/kg) on days 2, 5, and 8. Tumor volumes and body weight were measured throughout the study and calculated as previously described.

Flow cytometry-base immune cell analysis.

Tumor samples were prepared as previously described, and cells were subsequently stained with Fixable Viability Dye eFluor™ 780 (ThermoFisher Scientific). Initial gating was performed on live, singlet lymphocytes identified by forward scatter (FSC) and side scatter (SSC) parameters (using height, area, and width to exclude doublets), followed by gating on $CD45^+$ cells. Immune subsets were defined as follows: NK cells ($CD45^+NK1.1^+$), macrophages ($CD45^+F4/80^+$) and B cells ($CD45^+CD3^-CD19^+$). The list of antibodies and panel designations are provided in Tables S1 and S2.

Immunohistology.

IF staining: Tumor tissues slices were heated in Citrate Buffer (10 mM sodium citrate, 0.05% Tween-20, pH 6.0) at 95°C for 20 minutes, then blocked with 10% goat serum for 60 mins at room temperature. IF staining was performed using primary antibodies, followed by incubation with fluorescence-conjugated secondary antibodies. The antibody sets were following: for CD45 detection, Primary: Rabbit monoclonal anti-CD45 [EPR20033] (Abcam), Secondary: Goat Anti-Rabbit IgG H&L Alexa Fluor® 488 (Abcam); for CD69/CD3ε detection, Primary: Rabbit polyclonal anti-CD69 (Abcam) and Mouse monoclonal anti-CD3ε [EPR22667-12] (Abcam),

Secondary: Goat Anti-Rabbit IgG H&L Alexa Fluor® 594 (Abcam); for CD4/CD8 detection, Primary: Rat monoclonal anti-CD4 [GK1.5] (R&D Systems) and Rat monoclonal anti-CD8 [53-6.7] (Novus), Secondary: Goat Anti-Rat IgG H&L Alexa Fluor® 488 (Abcam). After washing, sections were counterstained with Hoechst 33342 (Beyotime) and mounted with coverslips. Images were acquired using an Olympus FV3000RS confocal microscope (Olympus).

H&E staining: At the end of experiment, liver and kidney tissues were harvested, fixed in 10% formalin, embedded in paraffin, and sectioned into 5-μm-thick slices. The tissue sections were deparaffinized in xylene and rehydrated through a graded series of ethanol solutions. The sections were then stained with hematoxylin and eosin using HE Staining System (KohyPath, Shanghai, China) according to the manufacturers' protocol. The slides were dehydrated, cleared, and mounted with coverslips. Images were acquired using an Olympus FV3000RS confocal microscope (Olympus)

Figures

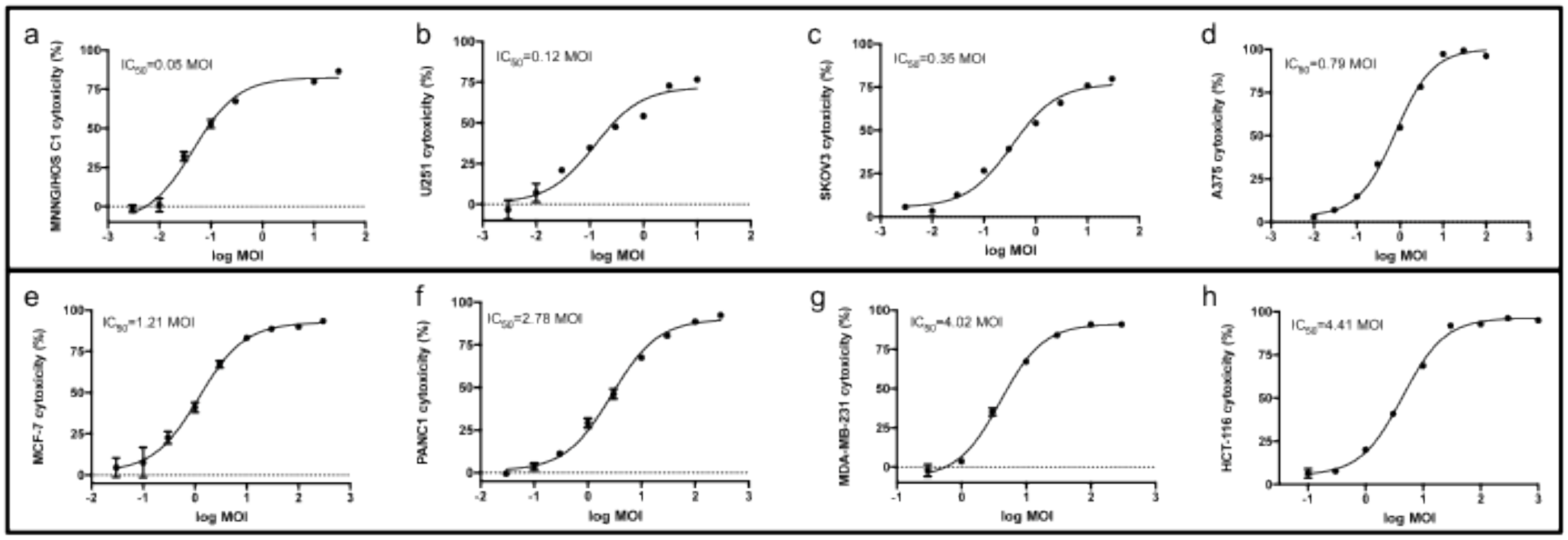

Figure S1. hV01 effectively inhibited the growth of various tumor cells. (a-h) Dose-dependent effects of hV01 on the cell viability of MNNG/HOS C1 (a), U251 (b), SKOV3 (c), A375 (d), MCF-7 (e), PANC1 (f), MDA-MB-231 (g), and HCT116 (h) cells after 48 hpi. Values were the means of three independent experiments, and data are shown as means ± SEM.

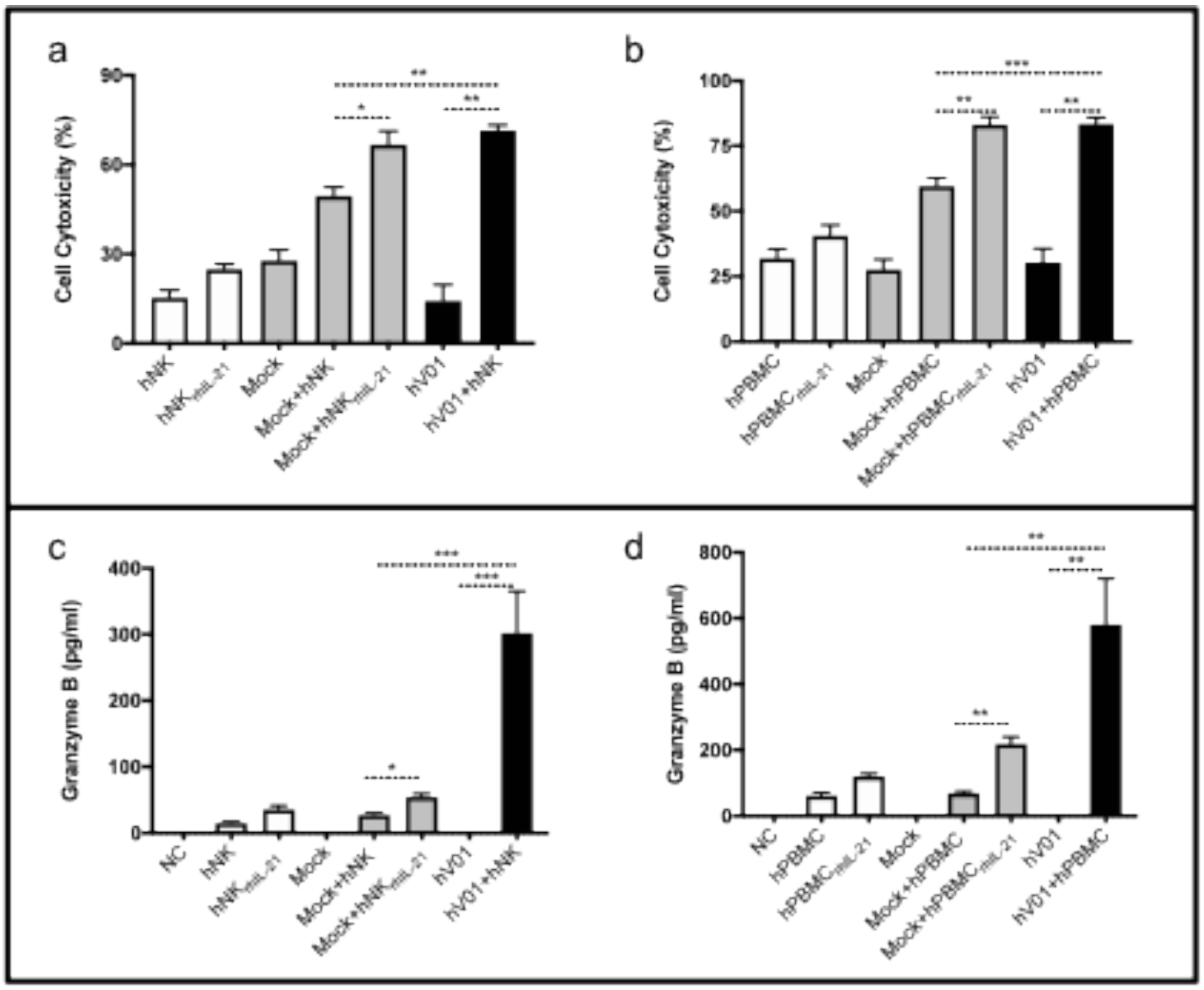

Figure S2. hIL-21 enhanced ICD in vitro. (a, b) Cell cytotoxicity and (c, d) granzyme B production were measured in MDA-MB-231 cells infected with hV01 or mock virus (MOI = 1.0) and co-cultured with hNK cells (a, c) or hPBMCs (b, d). Data represent the mean ± SEM of three independent experiments. Statistical significance was determined by one-way ANOVA: * $P < 0.05$; ** $P < 0.01$; *** $P < 0.001$.

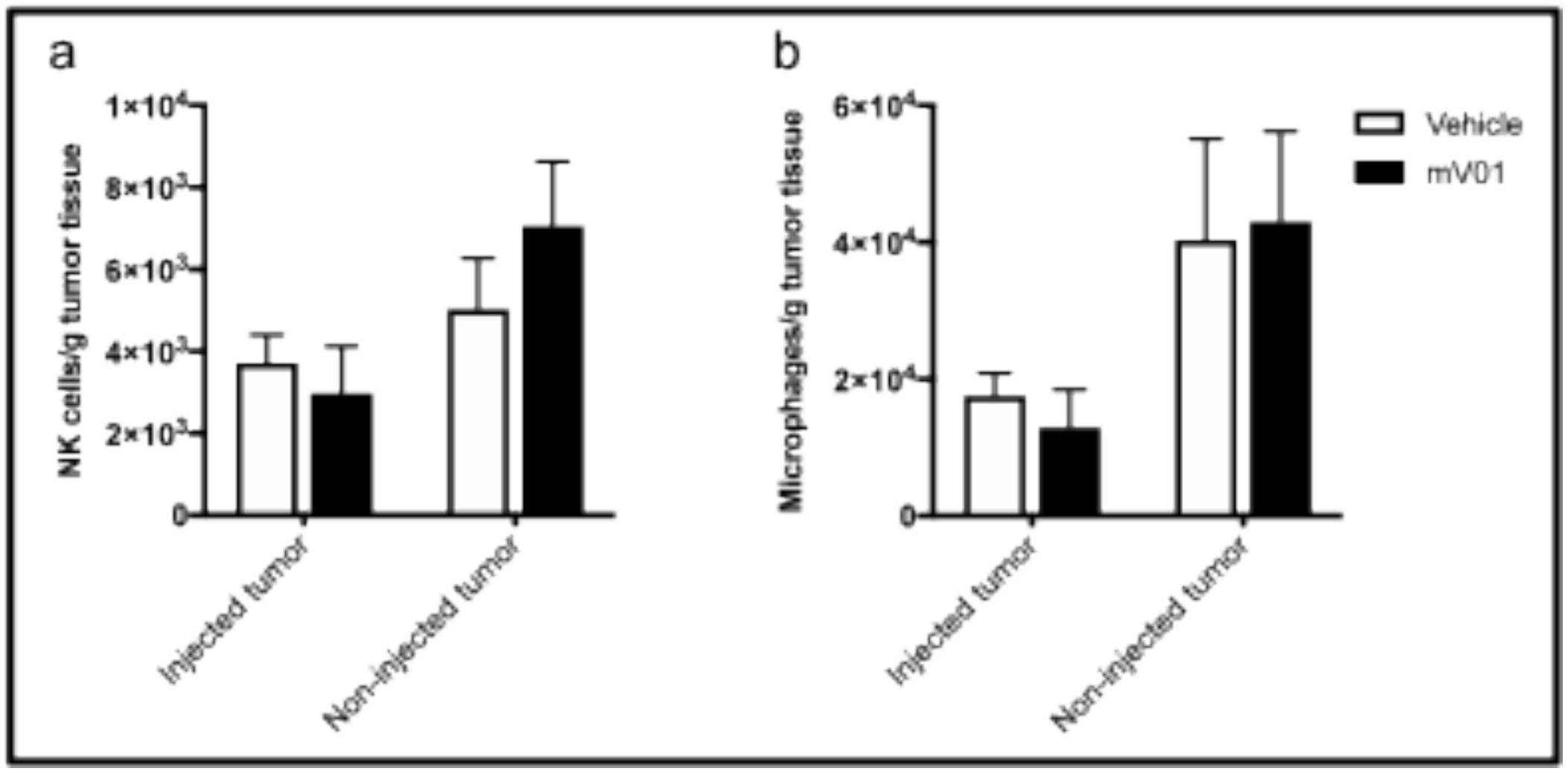

Figure S3. Quantitative analysis of immune cell populations of NK cell (a) and Microphage (b) on the injected tumors and non-injected tumors of B16 bilateral tumor-bearing mice. Vehicle, opened bars; mV01, closed bars. n = 5-6 mice/group. Data were presented as the mean ± SEM. Statistical significance was determined by an unpaired two-tailed Student's t-test.

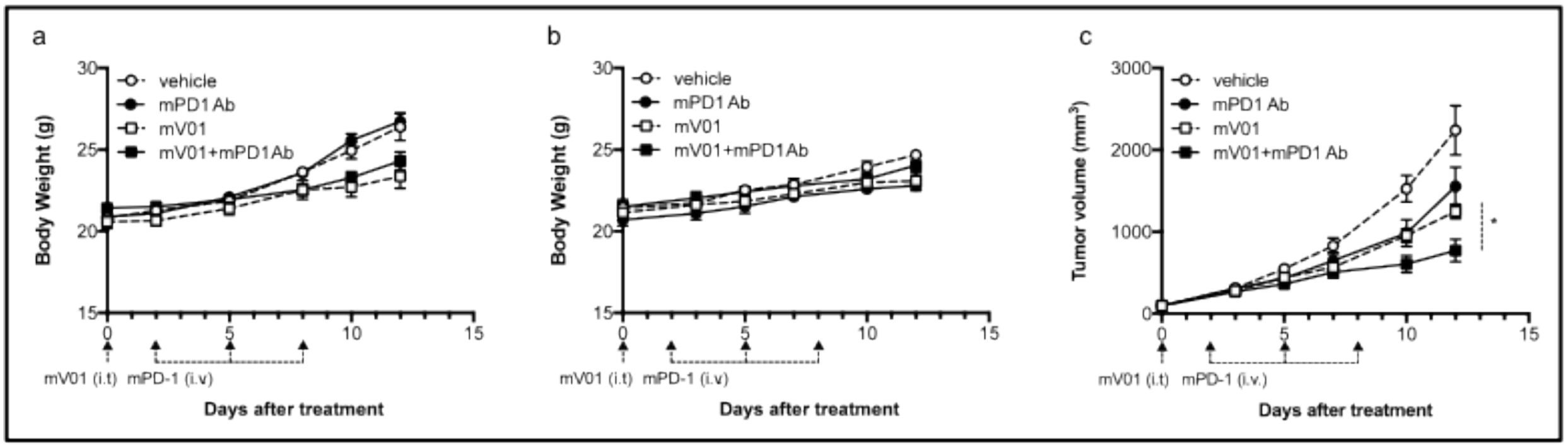

Figure S4. Effects of combination treatment on body weight and tumor progression. (a, b) Body weight of B16 tumor- (a) and MC38 tumor-bearing mice (b), and (c) tumor growth curve in MC38-bearing mice. Mice were treated with vehicle (open circles), mV01 (closed circles), anti–mPD-1 antibody (open squares), or the combination (closed squares). *n* = 8-10 mice/group. All values were presented as the mean ± SEM. **P* < 0.05 by two-way ANOVA test with Bonferroni correction.

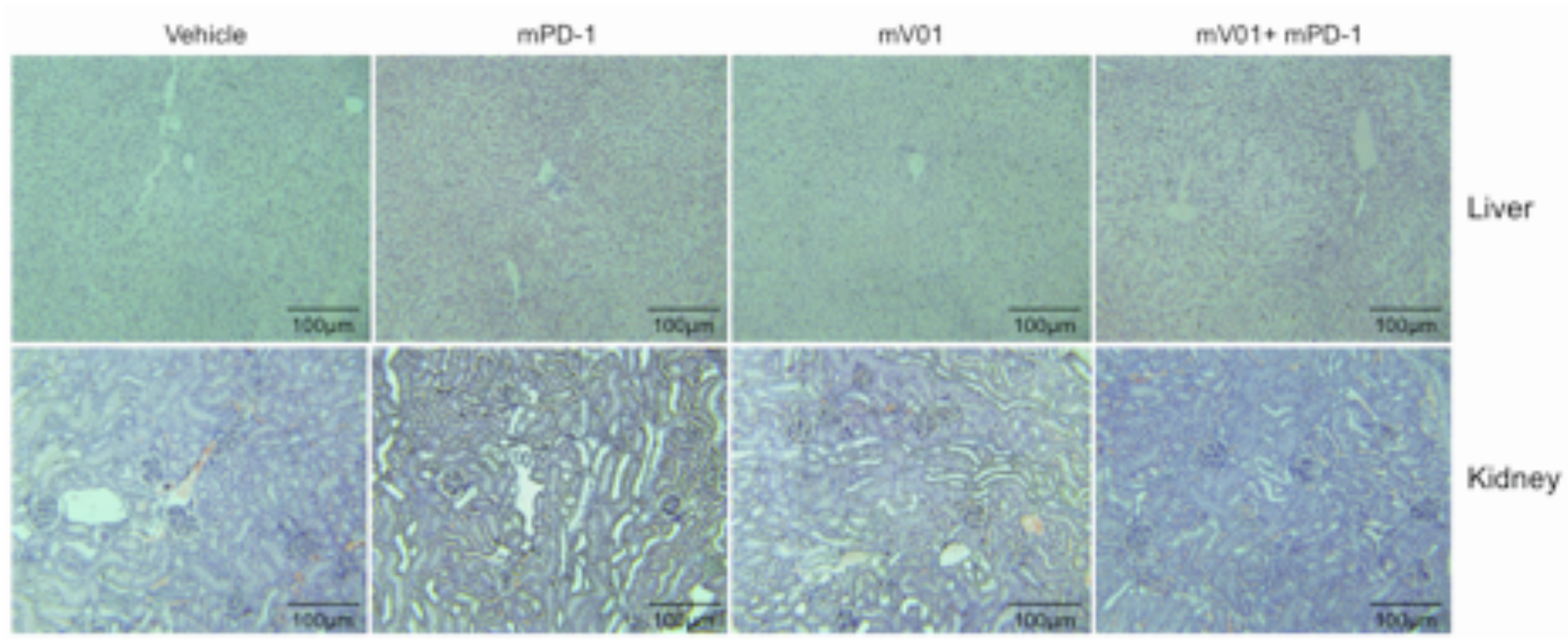

Figure S5. No toxicity effect of combination treatment in B16 tumor-bearing mice. H&E staining of Liver tissue (Top panels) and kidney tissue (bottom panels) of B16 tumor-bearing mice treated with vehicle, anti–mPD-1 antibody, mV01 and the combination. *n* = 8-10 mice/group. Scale bars: 100 μm.

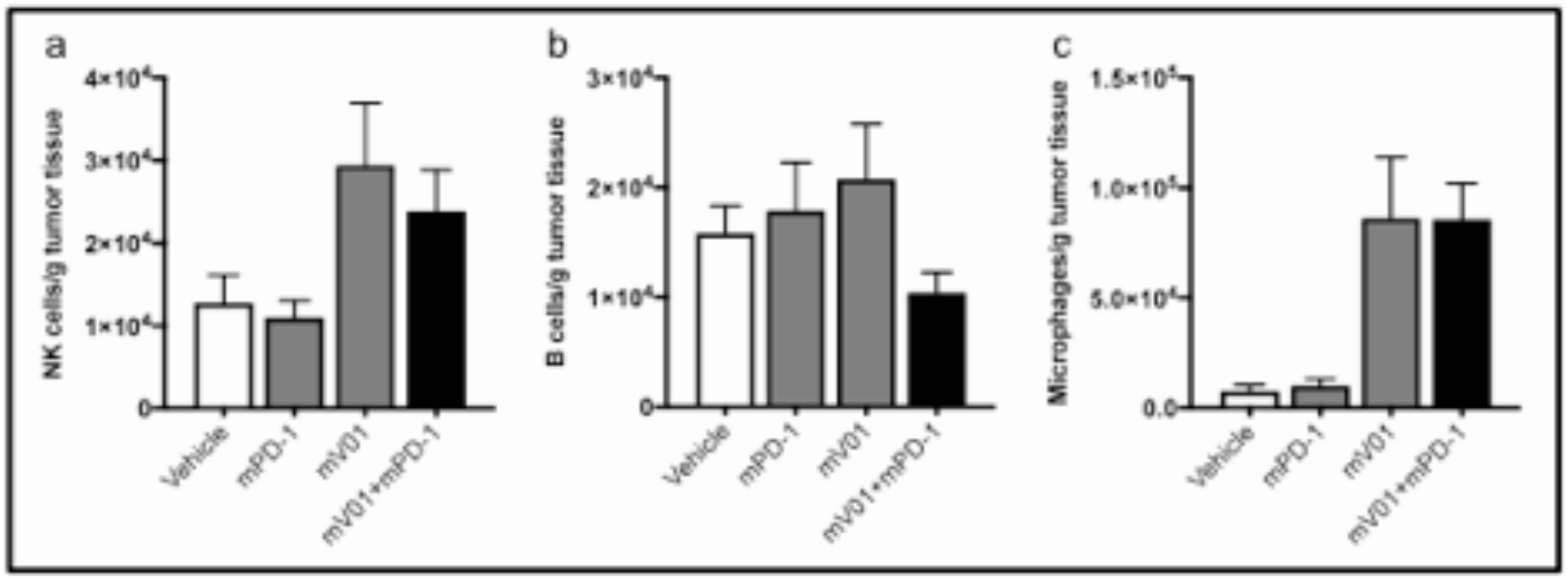

Figure S6. Quantitative analysis of (a) NK cells, (b) B cells and (c) Microphages, assessed by flow cytometry, in tumors from B16 tumor-bearing mice treated with vehicle, mV01, mPD-1 antibody and mV01 plus mPD-1 antibody. n = 10 mice/group. All values were presented as the mean ± SEM. Statistical significance was determined by one-way ANOVA.

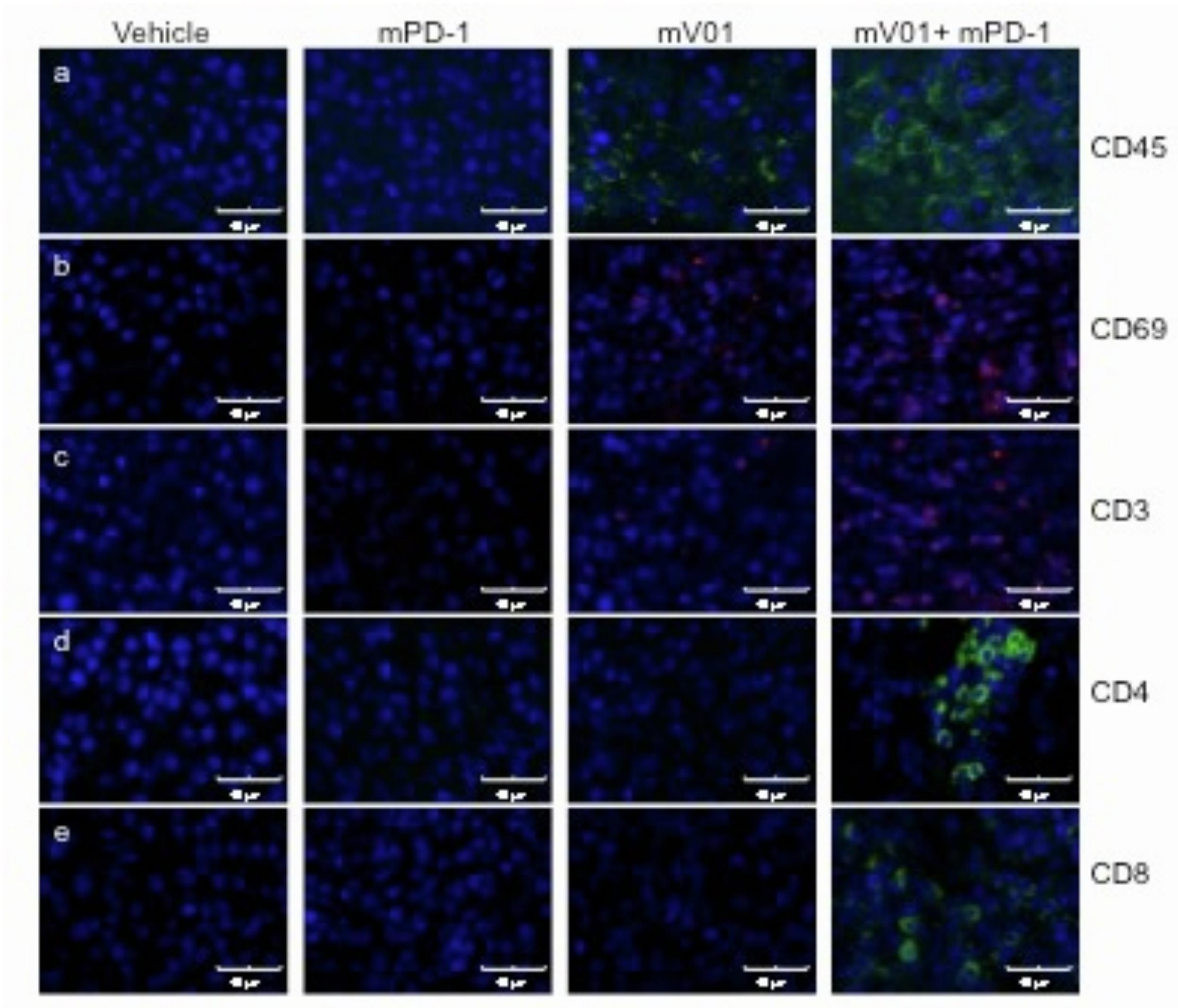

Figure S7. Blockage of PD-1 signal promoted V01-mediated anti-tumor immunity. IF staining was performed using antibodies against (a) CD45, (b) CD69, (c) CD3, (d) CD4 and (e) CD8 on the tumor sections from B16 tumor-bearing mice treated with vehicle, mPD-1 antibody, mV01, and mV01 plus mPD-1 antibody. Scale bars: 40 μm.

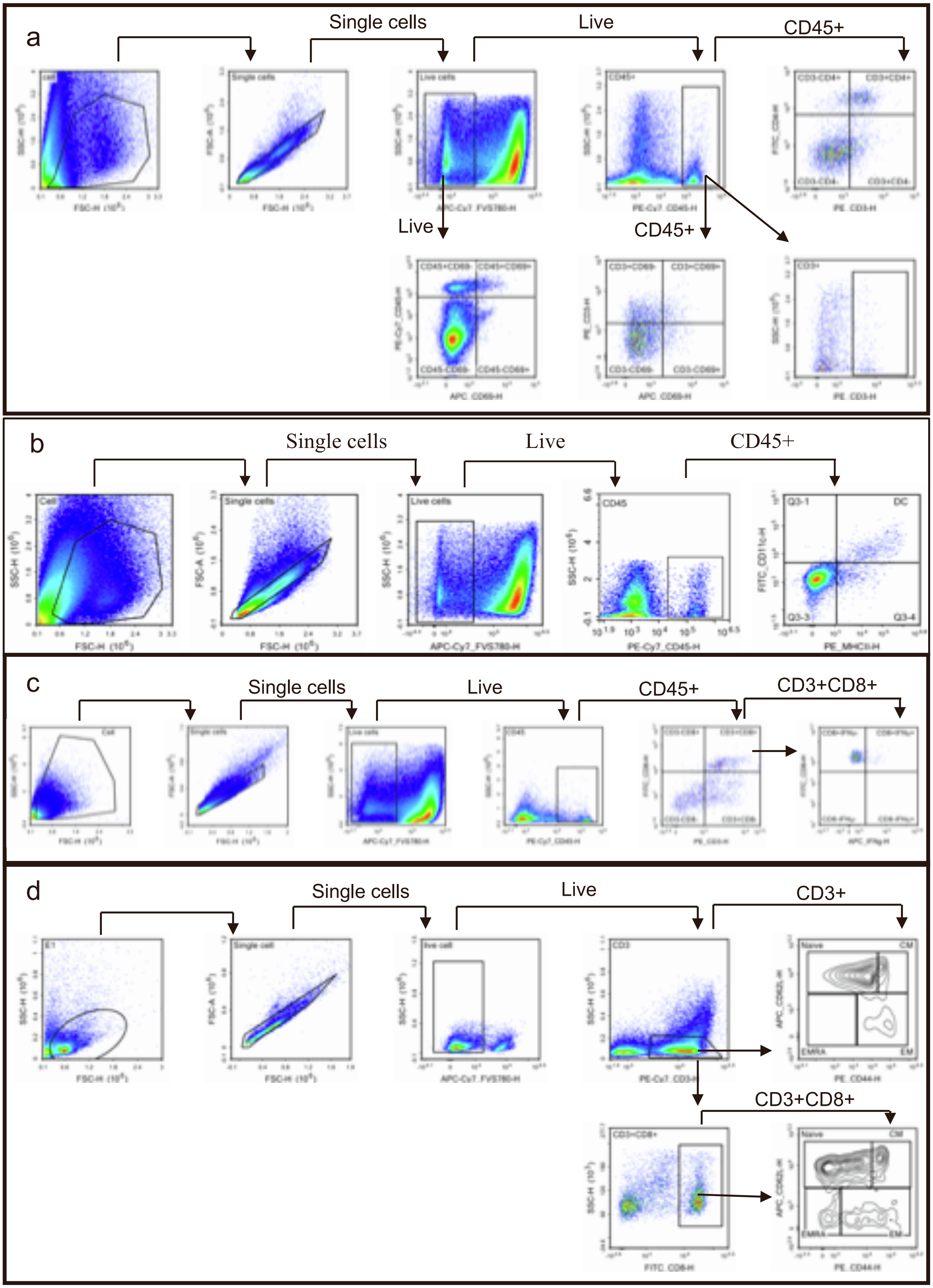

a
Single cells
Live
CD45+
Live
CD45+
b
Single cells
Live
CD45+
c
Single cells
Live
CD45+
CD3+CD8+
d
Single cells
Live
CD3+
CD3+CD8+

Figure S8. Gating strategy for (a) CD45+CD69+ active lymphocytes and CD45+CD3+CD69+ active T cells. In this example, CD45 PE-Cy7, CD3e PE, and CD69 APC were used. (b) CD45+CD11c+MHC II+ DC cell. In this example, CD45 PE-Cy7, CD11c FITC, and MHC II PE were used. (c) CD45+CD3e+IFN-γ+CD8a+/CD4+ IFN-γ+CD4/CD8 T cells. In this example, CD45 PE-Cy7, CD3e PE, CD8a FITC/CD4 FITC, and IFN-γ APC were used. (d) CD3+CD8+CD44-CD62L+ Naïve T cells, CD3+CD8+CD44+CD62L- TEM cells, CD3+CD8+CD44+CD62L+ TCM cells. In this example, CD3 PE-Cy7, CD8a FITC, CD44 PE, and CD62L APC were used.

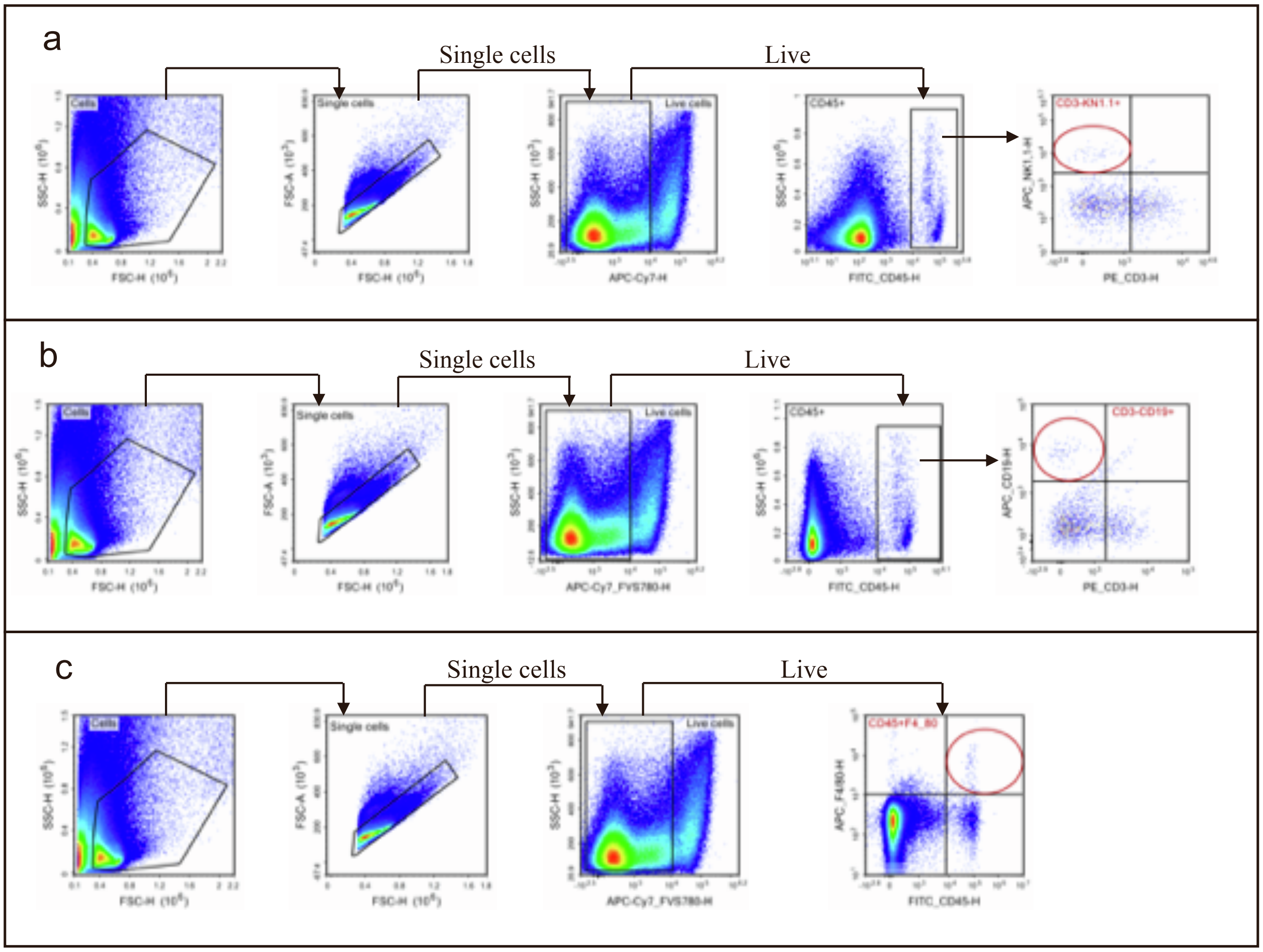

Figure S9. Gating strategy for (a) CD45+CD3-NK1.1+ NK cells. In this example, CD45 FITC, CD3e PE, and NK1.1 APC were used. (b) CD45+CD3-CD19+ B cells. In this example, CD45 FITC, CD3e PE, and CD19 APC were used. (c) CD45+F4/80+ microphages. In this example, CD45 FITC and F4/80 APC were used.

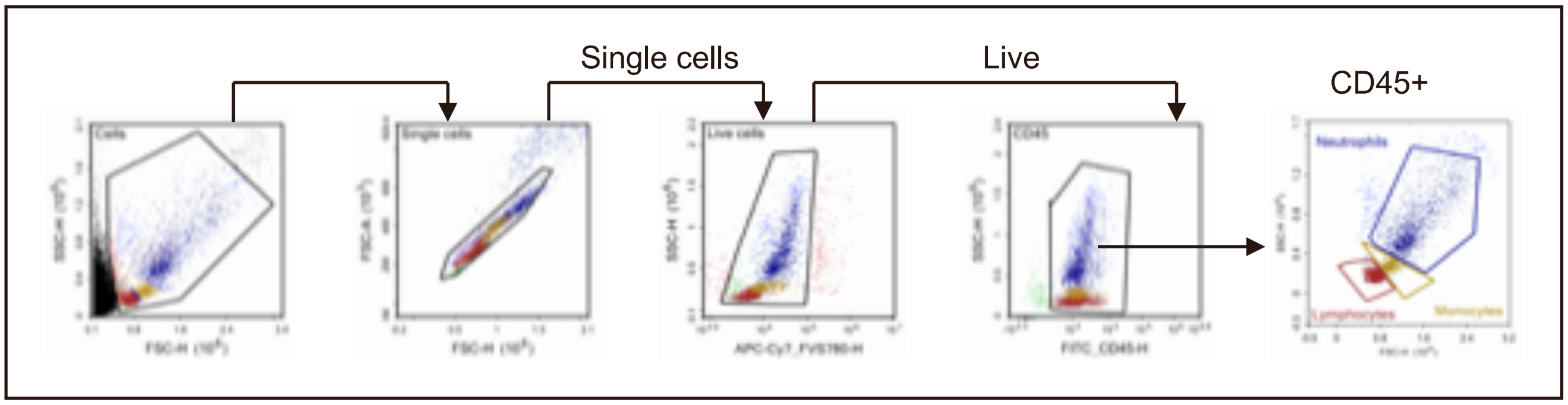

Figure S10. Gating strategy for hamster blood. The WBC fraction was incubated with FITC fluorescein-labelled anti-CD45 and subjected to a live/dead Fixable Viability Dye eFluor™ 780 staining. The major immune cell subsets, lymphocytes, monocytes and granulocytes, were gated according to their light-scattering characteristics.

Table S1 List of antibodies used in flow cytometry

Antigen	Clone	Fluorochrome	Vendor	Catalog#
CD45	30-F11	FITC	BD	553080
CD45	30-F11	PE-Cy7	eBioscience	25-0451-81
CD45	30-F11	APC	eBioscience	17-0451-82
CD11c	N418	FITC	Biolegend	117306
MHC II	NIMR-4	PE	eBioscience	12-5322-81
CD69	H1.2F3	APC	eBioscience	17-0691-82
CD3e	145-2C11	PE	eBioscience	12-0031-82
CD3	17A2	PE-Cy7	eBioscience	25-0032-82
CD4	RM4-5	FITC	eBioscience	11-0042-85
CD8a	53-6.7	FITC	Biolegend	100705
IFN-γ	XMG1.2	APC	Biolegend	505810
CD44	IM7	PE	eBioscience	12-0441-82
CD62L	MEL-14	APC	eBioscience	17-0621-82
CD86	GL1	FITC	eBioscience	25-0862-82
NK1.1	PK136	APC	BD	550627
F4/80	BM8	APC	eBioscience	17-4801-80
CD19	1D3	APC	BD	550992
CD45	HAT13A	--	Novus Biologicals	NBP2-60910
FITC Goat anti-mouse IgG (H+L)	--	FITC	Jackson	115-095-003

Table S2 Flow cytometry panel designation

Panel	Antigen	Clone	Fluorochrome
DC	CD45<br>CD11c<br>MHC II	30-F11<br>N418<br>NIMR-4	PE-Cy7<br>FITC<br>PE
CD45 activation	CD45<br>CD69	30-F11<br>H1.2F3	PE-Cy7<br>APC
T cell activation	CD45<br>CD3e<br>CD69	30-F11<br>145-2C11<br>H1.2F3	PE-Cy7<br>PE<br>APC
CD4 T cell	CD45<br>CD3e<br>CD4	30-F11<br>145-2C11<br>RM4-5	PE-Cy7<br>PE<br>FITC
CD8 T cell	CD45<br>CD3e<br>CD8a	30-F11<br>145-2C11<br>53-6.7	PE-Cy7<br>PE<br>FITC
IFNγ^{+}CD4 T cell	CD45<br>CD3e<br>CD4<br>IFN-γ	30-F11<br>145-2C11<br>RM4-5<br>XMG1.2	PE-Cy7<br>PE<br>FITC<br>APC
IFNγ^{+}CD8 T cell	CD45<br>CD3e<br>CD8a<br>IFN-γ	30-F11<br>145-2C11<br>53-6.7<br>XMG1.2	PE-Cy7<br>PE<br>FITC<br>APC
Memory T cell	CD44<br>CD62L<br>CD3<br>CD8a	IM7<br>MEL-14<br>17A2<br>53-6.7	PE<br>APC<br>PE-Cy7<br>FITC
NK Cell	CD45<br>CD3e<br>NK1.1	30-F11<br>145-2C11<br>PK136	FITC<br>PE<br>APC
Microphage	CD45<br>F4/80	30-F11<br>BM8	FITC<br>APC
B Cell	CD45<br>CD3e<br>CD19	30-F11<br>145-2C11<br>1D3	FITC<br>PE<br>APC
