# Supplementary material for: Interleukin-21 expressing oncolytic vaccinia virus elicits systemic anti-tumor immune response and synergizes with immunotherapies
Source: Mol Ther Oncol. 2026 Sep 18;34(4):201348. doi: 10.1016/j.omton.2026.201348 (PMC13720255; doi:10.1016/j.omton.2026.201348)
Supplement: Supplementary file 2 [file mmc2.pdf]

Molecular Therapy
Oncology
Original Article

Interleukin-21 expressing oncolytic vaccinia virus elicits systemic anti-tumor immune response and synergizes with immunotherapies

Rong Zhang,[1] Tingting Wang,[1] Yu Wang,[2] Chenggang Hong,[1] Yun Qin,[1] Kangchen Ye,[1] Hu Zhao,[2] Fang Hu,[1] Jinlu Cai,[1] and Jin Fu[1]

[1]Hangzhou Converd Co. Ltd., Hangzhou, China; [2]Department of General Surgery, Fuzong Clinical Medical College of Fujian Medical University, Fuzhou, China

A successful oncolytic virus is critical to therapeutic success, requiring the identification of an effective viral platform and immunomodulatory molecules that potently promote host immune responses and reprogram the tumor microenvironment (TME). In the present study, we developed V01, an oncolytic virus derived from the Western Reserve (WR) strain of vaccinia virus, featuring double deletions of the vaccinia growth factor (VGF) and thymidine kinase (TK) genes, along with the expression of interleukin-21 (IL-21). Locally administered intratumoral (i.t.) V01 elicited systemic anti-tumor effects against both the primary injected tumor and distant non-injected tumors in immunocompetent tumor-bearing mice. Abscopal effects of V01 were observed in homologous tumors but not in heterologous tumors. Combination treatment of V01 with immunotherapeutic agents—PD-1 antibody or natural killer (NK) cell therapy—exhibited synergistic tumor regression. Together, these results suggest that V01 holds significant promise as a novel immunotherapeutic or immunoadjuvant for solid tumor treatment, warranting further clinical investigation.

INTRODUCTION

Oncolytic viruses have emerged as a highly promising approach for cancer treatment by directly inducing viral lysis of tumor cells and harnessing the power of the host immune system. In the field of immune oncology, although immune checkpoint inhibitors (ICIs) and chimeric antigen receptor (CAR) T cell therapies have achieved clinical success in a small subset of cancer patients, a large proportion of patients still show no response or only minor responses to these immunotherapeutics.[1] The limited efficacy of these therapies is often attributed to the cold tumor phenotype, characterized by a paucity of tumor-infiltrating lymphocytes (TILs) and a highly immunosuppressive tumor microenvironment (TME).[2–4] Therefore, strategies aimed at converting cold tumors into hot tumors within the TME are crucial for overcoming the limitations of these immunotherapeutics in poorly responding tumors. Oncolytic viruses preferentially replicate in tumor cells and induce immunogenic cell death (ICD), which initiates tumor immunity through the release of tumor-associated antigens (TAAs) and damage-associated molecular patterns (DAMPs).[5,6] More importantly, intratumoral (i.t.) replication of oncolytic viruses promotes the activation of both the innate and adaptive immune systems, generating local inflammatory responses that negate TME immunosuppression and convert cold tumors into hot tumors.[7] Among the various types of oncolytic viruses that have undergone investigation,[5] vaccinia virus has one of the most extensive clinical experiences to date. In vaccinia virus, the endogenous genes encoding vaccinia growth factor (VGF) and thymidine kinase (TK) are commonly targeted for deletion to attenuate viral pathogenicity and restrict viral replication to tumor cells. This strategy exploits the unique physiology of tumor cells, which exhibit constitutive activation of the EGFR-Ras-MEK pathway and elevated E2F-driven deoxyribonucleotide triphosphate (dNTP) synthesis,[8,9] thereby creating conditions that compensate for the missing viral genes and support viral replication selectively within tumors.[10,11] To enhance tumor tropism and limit off-target toxicity, we engineered vaccinia virus with double deletions in the viral TK and VGF genes to functionally target the EGFR/VEGFR signaling pathways. These modifications attenuated viral pathogenicity in non-transformed cells while preserving viral potency against a broad panel of tumor cell lines.

Although oncolytic viruses have emerged as powerful immunostimulatory agents capable of activating and redirecting innate and adaptive immune responses against tumors, resistance to oncolytic virotherapy remains a significant challenge that may hamper clinical efficacy.[12–14] To overcome these hurdles and enhance therapeutic efficacy, cytokine-armed oncolytic viruses have emerged as a promising strategy. As a member of the common gamma-chain cytokine family, IL-21 has demonstrated potent anti-tumor activity, including the ability to regulate T cell functions,[15–17] facilitating natural killer (NK) cell expansion and reversing NK cell exhaustion,[18–20] with a favorable toxicity profile.[21,22] Although recombinant IL-21 protein

Received 15 December 2025; accepted 16 September 2026;
https://doi.org/10.1016/j.omton.2026.201348.

Correspondence: Jinlu Cai, Hangzhou Converd Co. Ltd., Hangzhou, China.
E-mail: caijinlu@converd.com.cn
Correspondence: Jin Fu, Hangzhou Converd Co. Ltd., Hangzhou, China.
E-mail: fujin@converd.com.cn

Published by Elsevier Inc. on behalf of The American Society of Gene and Cell Therapy.
This is an open access article under the CC BY license (http://creativecommons.org/licenses/by/4.0/).

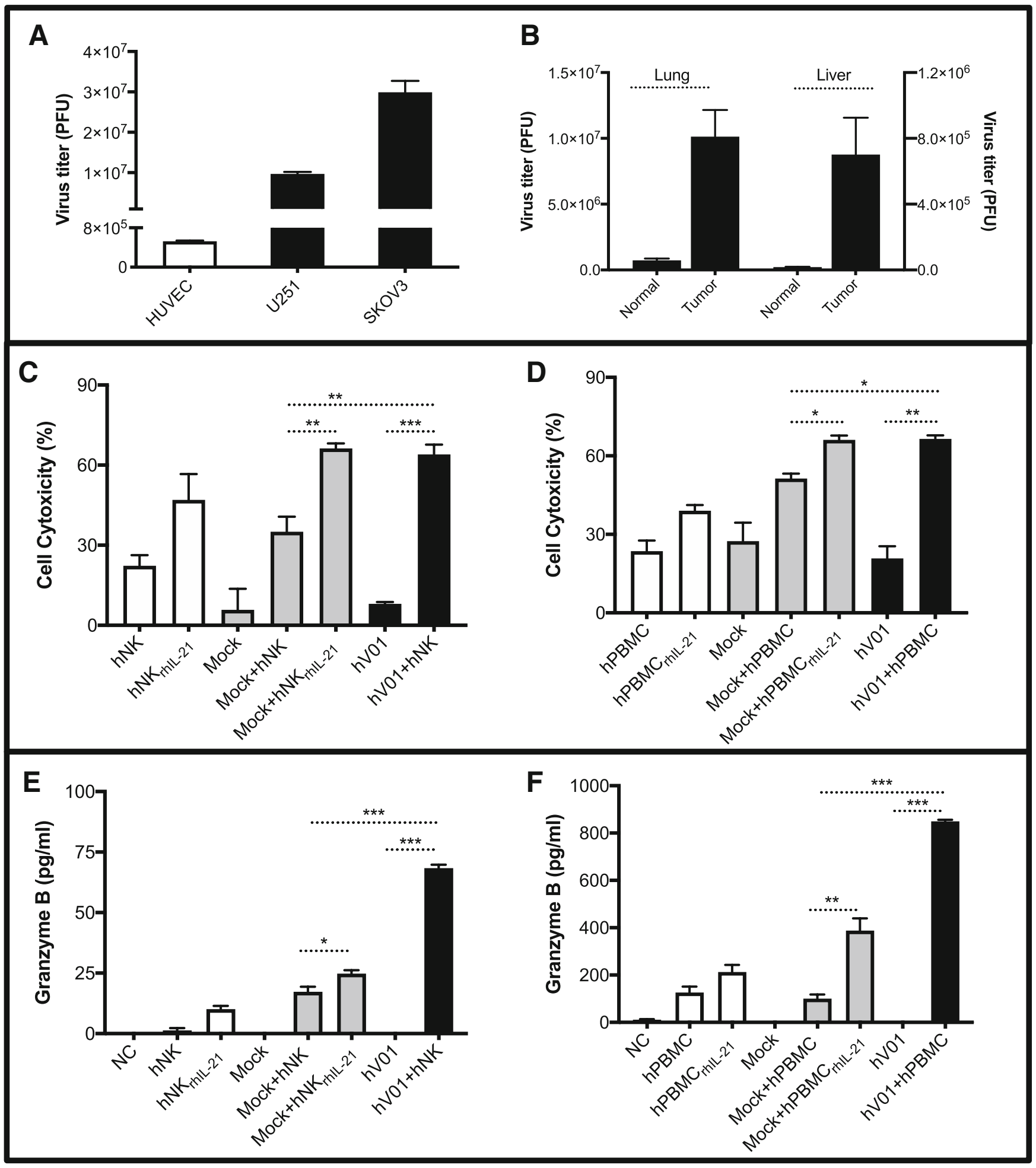

Figure 1. Selective replication of V01 in tumor tissues

(A) Viral replication kinetics were assessed by plaque assay in various human cell lines infected with hV01. (B) Viral titers in mouse normal tissues vs. tumor tissues following infection with mV01. (C–F) Cell viability (C and D) and granzyme B production (E and F) were measured in PANC-1 cells infected with hV01 or mock virus (MOI = 0.3) and co-cultured with hNK cells (C and E) or hPBMCs (D and F). Data represent the mean ± SEM of three independent experiments. Statistical significance was determined by one-way ANOVA: *$p < 0.05$; **$p < 0.01$; ***$p < 0.001$.

has been evaluated in clinical studies for cancer treatment, its rapid clearance and short half-life limit its clinical application.[23,24] Therefore, incorporating IL-21 into oncolytic viruses can mitigate these limitations by sustaining local IL-21 levels through viral replication, thereby enhancing anti-tumor effects via amplified immune stimulation.

Accumulating evidence supports that monotherapy is insufficient to control tumor progression and combination strategies are commonly used in the real world.[12,25] It has been hypothesized that the combination of oncolytic virotherapy with other immunotherapeutics, such as ICIs or cell therapy, could achieve additive or even synergistic effects.[26–29] In this study, we generated V01 variants expressing murine IL-21 (mV01) or human IL-21 (hV01) and evaluated their local and systemic antitumor efficacy. Immune profiling following local administration revealed that V01-induced systemic effects rely on TIL activation and specific tumor antigen recognition—a mechanism driving abscopal effects and long-term anti-tumor immunity. Furthermore, we elucidated the rationale for V01-based sequential combination therapies, demonstrating their potential to synergize with other immunotherapeutics against solid tumors.

RESULTS

V01 selectively replicates in tumors and promotes ICD

To assess the tumor selectivity of V01, we measured viral replication in non-transformed and transformed human cell lines, as well as in normal and tumor tissues from mice. In human cell lines, infection with hV01 at a multiplicity of infection (MOI) of 0.01 resulted in a dramatic increase in viral production in U251 and SKOV3 cells compared to that in human umbilical vein endothelial cell (HUVEC) cells at 48 h post-infection (hpi) (Figure 1A). Similarly, in an *ex vivo* organ culture study using lung and liver tissues from tumor-bearing and naive mice, mV01 production was markedly higher in tumor tissues than in normal tissues at 48 hpi with 1×10^2 PFU. Notably, mV01 exhibited minimal replication in normal tissues (Figure 1B), indicating its preferential oncolytic activity in tumors while sparing normal tissue.

Given its design for broad anti-tumor efficacy, hV01 demonstrated potent tumor cell killing across various human cell lines, with IC_{50} values ranging from 0.05 to 4.41 MOI (Figure S1). To evaluate the biological functions of the IL-21 encoded by hV01, we assessed immune-mediated cytotoxicity using human NK (hNK) cells and peripheral blood mononuclear cells (hPBMCs). Using the human pancreatic cancer cell line PANC-1 (Figures 1C–1F) and the breast cancer cell line MDA-MB-231 (Figure S2), we compared the effects of hV01 versus a mock virus in co-culture with hNK cells or hPBMCs. In PANC-1 cells, while hV01 and the mock virus at an MOI of 0.3 exhibited similar direct oncolytic efficacy, hV01-mediated killing was further enhanced in the presence of hNK cells at an effector-to-target (E:T) ratio of 1:1 (Figure 1C) or hPBMCs at an E:T ratio of 10:1 (Figure 1D). In contrast, mock virus-infected

cells showed minimal susceptibility to hNK cell- or hPBMC-mediated cytotoxicity. Notably, this cytotoxicity was significantly restored by the addition of exogenous recombinant human IL-21 (rhIL-21), mirroring the potent effects observed in hV01-infected co-cultures (Figures 1C and 1D). Furthermore, granzyme B release from hNK cells (Figure 1E) and hPBMCs (Figure 1F) was significantly elevated in both hV01-infected and rhIL-21-supplemented mock virus co-cultures, reinforcing the link between immune activation and tumor cell killing. Similar patterns were observed in MDA-MB-231 cells treated with either hV01 or mock virus in combination with hNK cells at an E:T ratio of 0.5:1 (Figures S2A and S2C) or hPBMCs at an E:T ratio of 5:1 (Figures S2B and S2E). Despite this rescue effect, granzyme B levels in the rhIL-21-supplemented mock group remained lower than those in the hV01-treated group. This indicates that while exogenous IL-21 can restore cytotoxicity, the sustained expression of IL-21 by hV01 overcomes rapid clearance and drives more robust immune activation.

IL-21 improves anti-tumor efficacy and enhances immune response

To elucidate the contribution of IL-21 expression to the efficacy of V01, we evaluated the anti-tumor effect of mV01 relative to a mock virus, with and without recombinant murine IL-21 (rmIL-21). In B16 melanoma tumor-bearing immunocompetent C57BL/6N mice, i.t. injections of mV01 on days 0, 2, and 4 resulted in a significantly greater reduction in tumor burden than that observed with the mock virus regimen. Notably, the therapeutic effect of the mock virus was significantly enhanced to a level comparable to that of mV01 upon the by the addition of exogenous rmIL-21 (Figure 2A), confirming that IL-21 secretion by mV01 contributes to its therapeutic efficacy.

ELISA analysis demonstrated significantly elevated levels of high-mobility group box 1 (HMGB1), a marker of tumor antigen release, in the tumor tissues of mV01-treated mice compared to those in the mock control. Consistent with the tumor growth inhibition results, treatment with rmIL-21 combined with the mock virus partially restored HMGB1 levels to those observed in the mV01 group (Figure 2B). Flow cytometry analysis revealed increased i.t. dendritic cell (DC) populations in the mock virus, mock virus plus rmIL-21, and mV01 treatment groups relative to untreated controls (Figure 2C), although no significant differences were observed among the treatment groups.

Flow cytometry analysis of TILs revealed an increase in $CD45^+$ lymphocytes, specifically the $CD69^+$-activated phenotype, across all virus-treated groups. Notably, a significant elevation was observed in the mV01 group compared to the mock virus treatment (Figure 2D). Although the increase in $CD45^+$ lymphocytes and the $CD69^+$ phenotype in the mock plus rmIL-21 group did not reach statistical significance compared to the mock group, a clear upward trend was observed, approaching the levels seen in the mV01 treatment group (Figure 2D). Mirroring the pattern observed in $CD45^+$ lymphocytes, $CD45^+CD3^+$ total T cells and the $CD3^+CD69^+$-activated phenotype exhibited similar trends (Figure 2E). T cell subset analysis showed increased proportions of $CD8^+$ T cells in all virus-treated groups, with a non-significant trend of increasing levels from mock to mock plus rmIL-21 and mV01 treatments. In contrast, the proportion of $CD4^+$ T cells remained unchanged across these groups (Figure 2F). Furthermore, intracellular cytokine staining revealed that the frequency of IFN-γ-secreting $CD8^+$ T cells was significantly higher in the mV01-treated and mock plus rmIL-21-treated groups compared to the mock-treated group (Figure 2G). Conversely, no significant difference in IFN-γ-secreting $CD4^+$ T cells was observed between the mV01-treated and mock-treated groups (Figure 2H), suggesting that IL-21 expression predominantly enhances $CD8^+$ T cell effector responses.

In addition, we characterized the immune profile in tumor-draining lymph nodes (TDLNs) following locally administered rmIL-21. Flow cytometry analysis revealed an increase in DC populations initiating on day 7 after the first virus administration in all virus-treated groups. However, while DC levels returned to baseline by day 11 in the mock-treated group, whereas they remained elevated in the rmIL-21 plus mock-treated and mV01-treated groups (Figure 2I). The proportion of total T cells within the live $CD45^+$ population was significantly increased in the rmIL-21 plus mock-treated and mV01-treated groups relative to the mock-treated group (Figure 2J). Specifically, this expanded T cell population primarily exhibited a CD44hiCD62L$^-$ phenotype, indicative of effector memory T cells, whereas the CD44hiCD62L$^+$ central memory phenotype remained unchanged (Figure 2J). Notably, this increase in effector memory T cells was accompanied by a concurrent rise in $CD8^+$ T cells with a $CD44^{hi}CD62L^-$ phenotype (Figure 2K). Together, these data suggest that IL-21 plays a crucial role in the anti-tumor efficacy and immune response elicited by the V01 oncolytic virus.

The abscopal effect of V01 is mediated by immune activation

The systemic effect of oncolytic viruses has been well elucidated, indicating the advantage of oncolytic virotherapy for treating both primary tumor and metastatic tumor.[12,26] To evaluate the therapeutic efficacy of mV01 in tumor control and systemic immune stimulation, we employed a syngeneic bilateral tumor model by implanting B16 melanoma cells into both flanks of immunocompetent C57BL/6N mice. When tumor volumes reached approximately 100 mm^3 (day 0), the right-flank tumors received three i.t. injections of mV01 at a dose of 1×10^6 PFU on days 0, 2, and 4, and tumor growth in both flanks was monitored. Day 10 after the first regimen, mV01-treated mice exhibited a significant reduction in tumor burden in both the injected and non-injected tumors compared with the vehicle-treated group (Figure 3A). ELISA analysis of tumor tissues revealed markedly increased levels of granzyme B (Figure 3B) and IFN-γ (Figure 3C) in both the injected and non-injected tumors of mV01-treated mice, with significantly higher levels observed in the injected tumors (Figures 3B and 3C). In contrast, elevated IL-21 levels were only observed in mV01-injected tumors, whereas levels in non-injected distant tumors remained comparable to those in

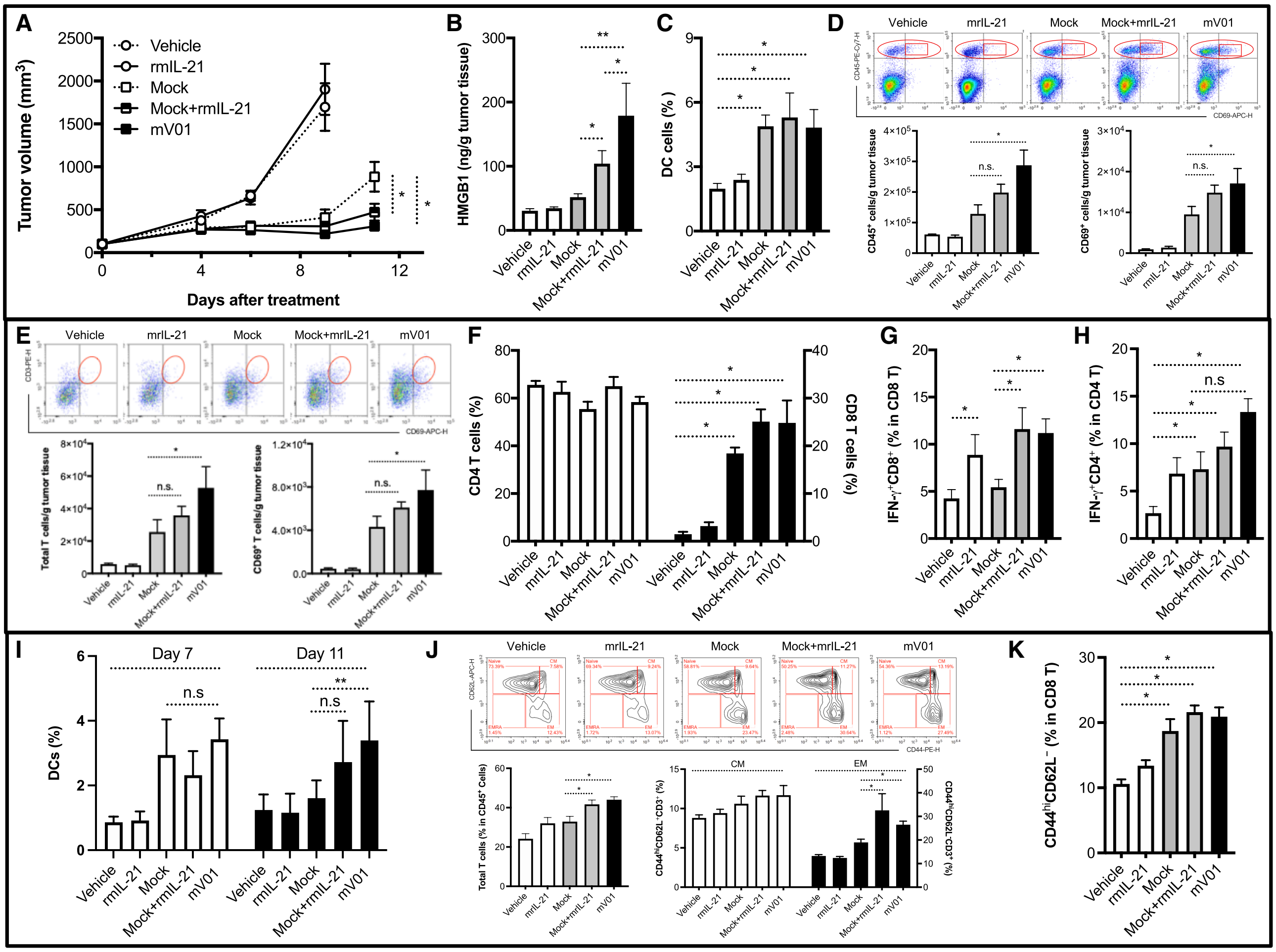

Figure 2. IL-21 enhances the therapeutic efficacy and immune response of V01

B16 tumor-bearing mice were treated with three intratumoral (i.t.) injections of mock virus (with or without rmIL-21) or mV01 on days 0, 2, and 4. (A) Tumor growth curves over time. (B) HMGB1 levels in tumor tissues. (C–H) Quantitative analysis of immune infiltration in tumor tissues, including the frequency of DCs (C), CD45+ lymphocytes and their activated phenotype (D), total T cells and their activated phenotype (E), the proportion of CD4+ and CD8+ T cells (F), and the frequency of IFN-γ-producing CD8+ T cells (G) and CD4+ T cells (H). (I–K) Quantitative analysis of immune populations in tumor-draining lymph nodes (TDLNs), showing the proportion of DCs (I), total T cells and memory phenotypes (J), and central memory CD8+ T cells (K). n = 5–6 mice per group. Data are presented as mean ± SEM. Statistical significance was determined using two-way repeated-measures ANOVA with Bonferroni post hoc correction (for tumor volumes) and one-way ANOVA (for ELISA and flow cytometry data): $^*p < 0.05$; $^{**}p < 0.01$; n.s., not significant.

the vehicle-treated group (Figure 3D). This finding suggests that the anti-tumor effect of mV01 at non-injected distant sites is independent of direct viral lysis, supporting the hypothesis of an immune-mediated systemic effect.

Harnessing TME immune cell responses is a crucial aspect of tumor immunotherapy. We analyzed the TIL profile following mV01 administration to elucidate the mechanisms driving the systemic response. Compared to vehicle-treated controls, mV01-treated mice exhibited robust lymphocyte recruitment in both injected and non-injected tumors (Figure 4). These recruited cells were identified as $CD45^{hi}CD69^+$ (Figure 4A), $CD45^+CD3^+$, $CD45^+CD3^+CD4^+$ (Figure 4B), $CD45^+CD3^+CD8^+$ (Figure 4C), and $CD45^+CD86^+$ (Figure 4D). Quantitative analysis confirmed a significant increase in $CD45^+$ lymphocytes (Figure 4E), which displayed an activated phenotype ($CD69^+$; Figure 4F), alongside significant expansions in total T cells (Figure 4G), $CD4^+$ T cells (Figure 4H), and $CD8^+$ T cells (Figure 4I). Notably, distant non-injected tumors in mV01-treated mice showed even higher infiltration of these immune subsets compared to the injected tumors (Figures 4E–4H). Furthermore, the frequency of $CD86^+$ antigen-presenting cells (APCs) increased in injected tumors, whereas no change was observed in non-injected tumors (Figure 4J). In contrast, NK cell and F4/80$^+$ macrophage frequencies remained unchanged in both tumor sites (Figures S3A and S3B). These findings suggest that local mV01 administration instigates a systemic immune response, priming T cell immunity via

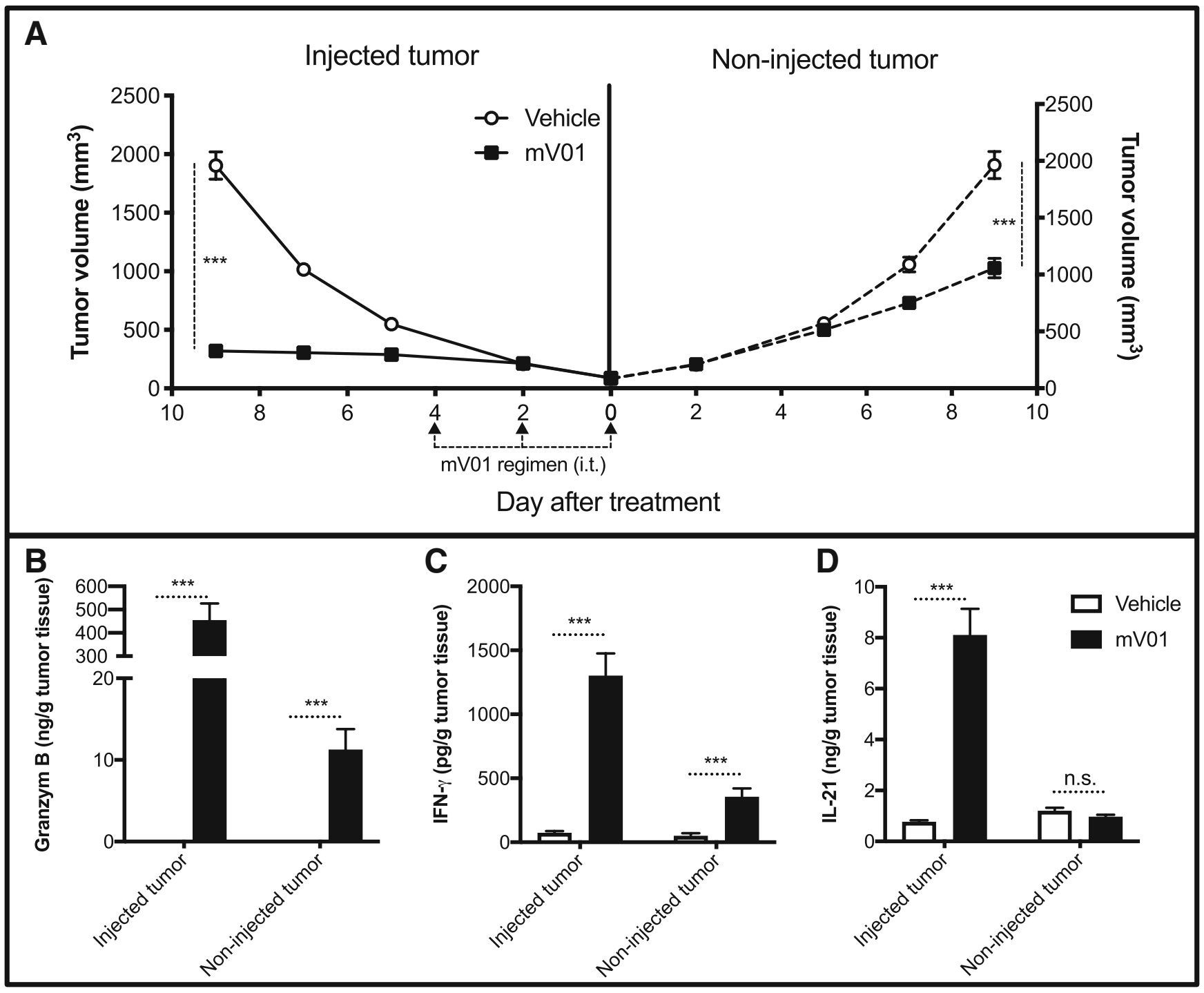

Figure 3. Local administration of mV01 reduces distant tumor burden in immunocompetent mice

B16 bilateral tumor-bearing mice received three i.t. injections of mV01 or vehicle into the right-flank tumors on days 0, 2, and 4. (A) Growth curves of injected and distant non-injected tumors. (B–D) Quantification of granzyme B (B), IFN-γ (C), and IL-21 (D) levels in tumor tissues, assessed by ELISA. n = 5–6 mice per group. Values represent mean ± SEM. Statistical significance was determined by two-way repeated-measures ANOVA with Bonferroni post hoc correction (for tumor volumes) and an unpaired two-tailed Student's t test (for ELISA assay): ***$p < 0.001$; n.s., not significant.

primary tumor antigens and contributing to the abscopal anti-tumor effect at distant non-injected tumors.

The abscopal effect of V01 is mediated by specific tumor antigen recognition

Previous findings demonstrated that local administration of mV01 profoundly suppressed tumor growth and effectively triggered systemic immunity. To further investigate whether the release of tumor antigens contributes to immune-mediated anti-tumor effects on distant tumors, we employed two murine tumor models: an EMT6/EMT6 homologous model and an EMT6/A20 heterologous model. In these models, BALB/c immunocompetent mice were implanted with EMT6 cells in both the right and left flanks (EMT6/EMT6 homologous) or with EMT6 cells in the right flank and A20 cells in the left flank (EMT6/A20 heterologous). As illustrated in the experimental design (Figure 5A), right-flank EMT6 tumors received three i.t. injections of mV01 at a dose of 1×10^6 PFU on days 0, 3, and 6, followed by contralateral rechallenge with either EMT6 or A20 cells on day 7. In the EMT6/EMT6 homologous mice, mV01 i.t. administration significantly reduced tumor burden not only in the injected tumors but also in the contralateral EMT6-rechallenged tumors (Figure 5B). Conversely, in the EMT6/A20 heterologous mice, inhibition of tumor growth was observed only in the mV01-injected tumors, whereas the contralateral A20-rechallenged tumors remained unaffected (Figure 5C).

Consistent with the observed tumor suppression, flow cytometric analysis of tumor tissues revealed a concurrent, at least partial, increase in TIL populations, including $CD3^+$ total T cells, $CD3^+CD4^+$ T cells, and $CD3^+CD8^+$ T cells, in both the mV01-injected and contralateral EMT6-rechallenged tumors of EMT6/EMT6 homologous mice (Figures 5D and 5F). In contrast, in the EMT6/A20 heterologous mice, TIL infiltration increased only at the mV01-injected site, with no change observed in the contralateral A20-rechallenged tumor (Figures 5E and 5G). Together, these results indicate that the distant anti-tumor effect of local mV01 administration is mediated by the priming of immune responses against tumor antigens specifically recognized by the host immune system in the context of the parental tumor.

V01 evokes long-term anti-tumor immunity

To investigate whether V01-mediated anti-tumor immunity involves the establishment of long-term immune protection, we assessed tumor growth following rechallenge in cured animals that had undergone V01 virotherapy. As illustrated in the experimental design (Figure 6A), LVG hamsters bearing Hap-T1 hamster pancreatic tumors received three i.t. injections of mV01 at a dose of 1×10^7 plaque-forming unit (PFU) on days 0, 3, and 6. Upon complete tumor clearance, the tumor-free animals were rechallenged with Hap-T1 cells to evaluate protective immunity. By day 29 post-treatment, primary Hap-T1 tumors in the mV01-treated group had achieved a complete response (Figure 6B). When rechallenge with 2×10^5 Hap-T1 cells on the contralateral flank, mV01-pretreated animals exhibited robust resistance to secondary tumor development. In contrast, vehicle-treated controls developed palpable tumors by day 7 post-rechallenge, with rapid progression to a volume of approximately 4,000 mm^3 by day 30 (Figure 6C). Together, these data suggest that mV01-induced systemic immune activation sustains long-term anti-tumor immunity.

Flow cytometric analysis of peripheral blood showed a significant increase in the lymphocyte population within the white blood cell (WBC) compartment in mV01-treated animals compared with vehicle-treated controls, whereas granulocyte frequencies decreased and monocyte frequencies remained unchanged (Figure 6D). To further characterize the specificity of mV01-elicited immunity, we

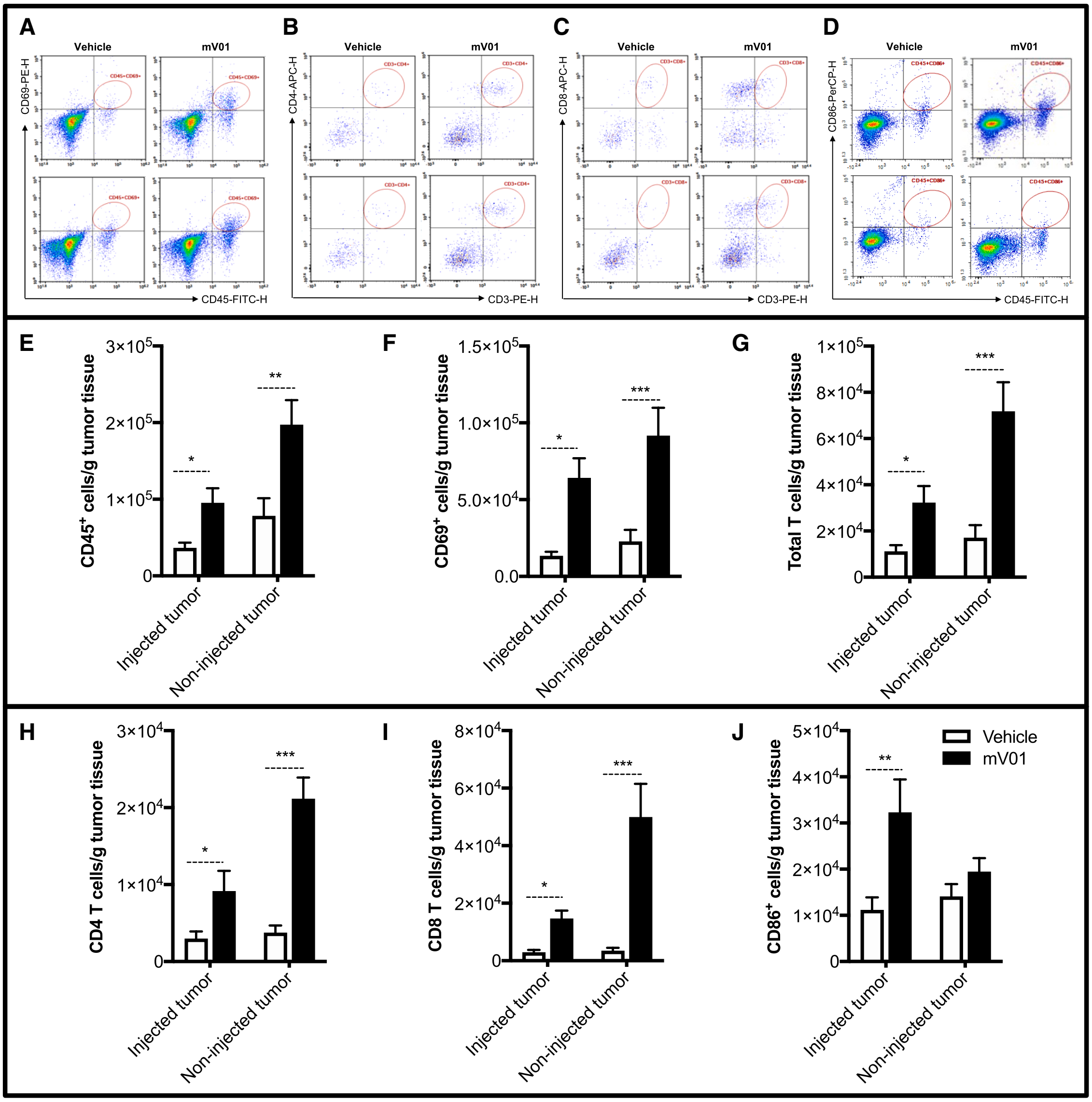

Figure 4. Local administration of mV01 recruits lymphocytes into TME of bilateral tumors

(A–C) Representative flow cytometry plots from injected (top) and non-injected (bottom) tumors of B16 bilateral tumor-bearing mice treated with vehicle or mV01. Plots show staining for CD45 and CD69 (A). Gating strategy on $CD45^+$ cells to identify total T cells ($CD3^+$), $CD4^+$ T cells (B), $CD8^+$ T cells (C), and $CD86^+$ APCs (D). (E–J) Quantitative analysis of the frequency of $CD45^+$ lymphocytes (E), $CD69^+$ activated lymphocytes (F), total T cells (G), $CD4^+$ T cells (H), $CD8^+$ T cells (I), and $CD86^+$ APCs (J) in both injected and distant non-injected tumors. n = 5–6 mice per group. Values represent mean ± SEM. Statistical significance was determined by an unpaired two-tailed Student's t test: $^*p < 0.05$; $^{**}p < 0.01$; $^{***}p < 0.001$.

performed an *in vitro* cytotoxicity assay using splenocytes derived from vehicle- or mV01-treated Hap-T1 tumor-bearing animals. When co-cultured with syngeneic Hap-T1 cells, splenocytes from mV01-treated animals demonstrated significantly stronger cytotoxic activity than those from vehicle-treated animals, whereas no significant difference was observed when co-cultured with Hak hamster kidney cells (Figure 6E). The concurrent increase in peripheral lymphocytes and the potent tumor-specific cytotoxic activity in mV01-treated animals are consistent with the sustained anti-tumor effects.

V01 synergizes with PD-1 blockade to enhance anti-tumor activity

Many studies have demonstrated that oncolytic viruses remodel the TME by recruiting and activating effector T cells, thereby amplifying ICD against tumors.[7] To enhance the anti-tumor efficacy of V01 virotherapy, we evaluated its combination with ICIs in B16 tumor-bearing mice, a poorly immunogenic model typically resistant to PD-1 antibody monotherapy. B16 tumor-bearing mice receiving the combination of mV01 with mPD-1 antibody exhibited

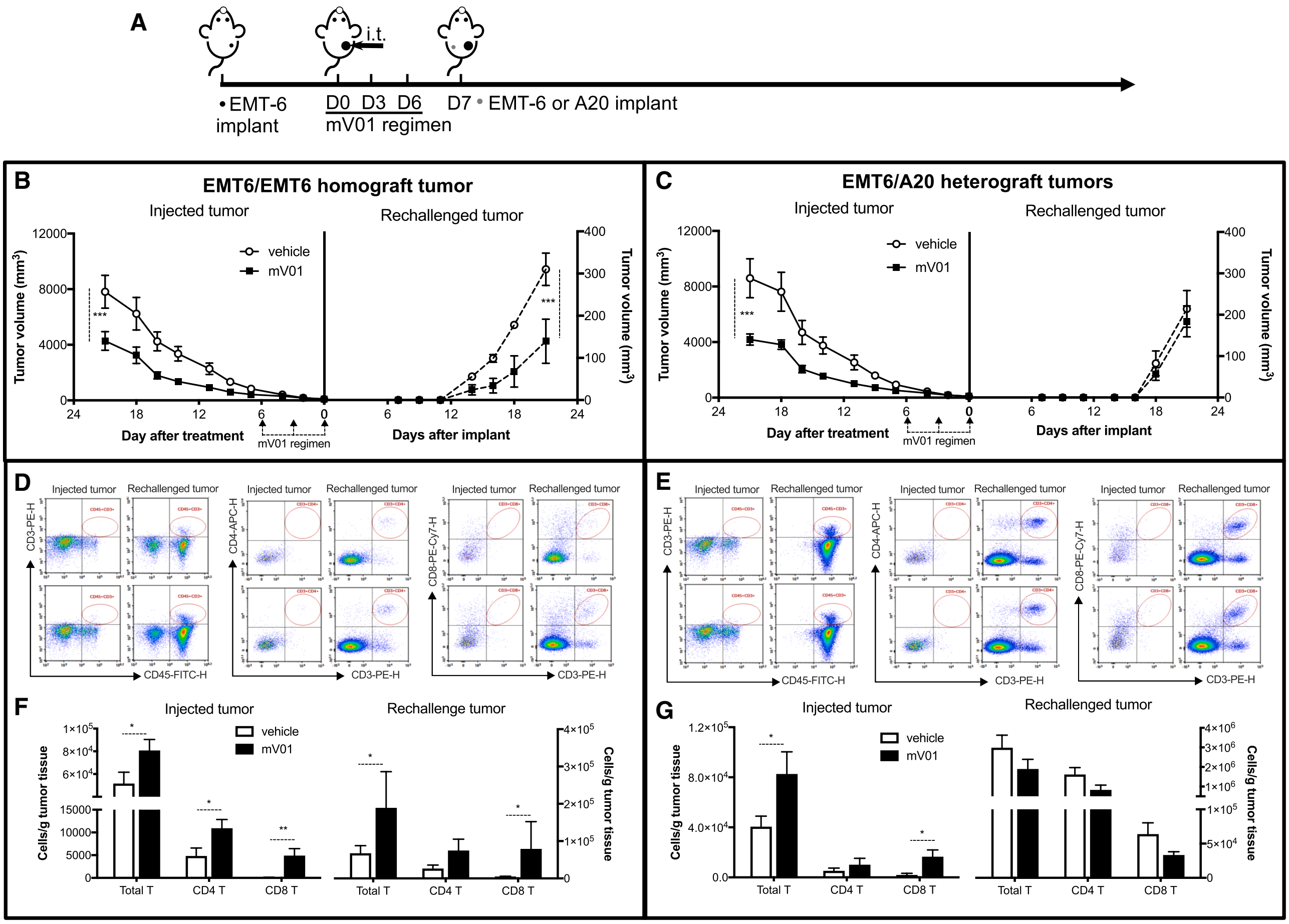

Figure 5. Systemic anti-tumor effects of local mV01 administration depend on tumor antigen recognition

(A) The scheme of experimental design. (B and C) Tumor growth curves for primary (injected) and secondary (rechallenged) tumors in mice bearing EMT6/EMT6 homografts (B) or EMT6/A20 heterografts (C). (D and E) Representative flow cytometry plots from primary and secondary tumors of EMT6/EMT6 (D) and EMT6/A20 (E) models treated with mV01 (top) or vehicle (bottom). Plots show gating for $CD45^+/CD3^+$ (total T cells), $CD3^+/CD4^+$ (CD4 T cells), and $CD3^+/CD8^+$ (CD8 T cells). (F and G) Quantitative analysis of immune cells in tumor tissues of EMT6/EMT6 (F) and EMT6/A20 mice (G). $n = 10$ mice per group. Values are mean ± SEM. Statistical significance was determined by two-way ANOVA with Bonferroni post-hoc correction (for tumor volumes) and an unpaired two-tailed Student's *t* test (for flow cytometry data): $^*p < 0.05$; $^{**}p < 0.01$; $^{***}p < 0.001$.

significantly greater tumor suppression compared to those receiving either mV01 or mPD-1 antibody monotherapy (Figure 7A), while maintaining stable body weights (Figure S4A). This enhanced anti-tumor efficacy was characterized by elevated levels of inflammatory modulators within the TME, including IFN-γ, granzyme B, TNF-α, and IL-6 (Figures 7B–7E), with no evidence of hepatic or renal toxicity (Figure S5). Moreover, the therapeutic benefits of this combination were recapitulated in MC38 colon tumor-bearing mice (Figures S4B and S4C), suggesting that mV01 virotherapy sensitizes tumors to PD-1 antibody by reprogramming the TME.

Given that i.t. immune activity is a key determinant of response to virotherapy, we analyzed TIL components and mV01 content within tumors. In B16 tumor-bearing mice, the combination of mV01 and mPD-1 antibody led to an increase in $CD45^+$ immune cell infiltration, characterized by a higher proportion of the $CD69^+$-activated phenotype (Figure 7F). Among the expanding TIL populations, we observed an enrichment of $CD45^+CD3^+$ total T cells, including both $CD4^+$ and $CD8^+$ T cell subsets (Figure 7G). In contrast, no significant changes were detected in NK cells, B cells, or macrophages as assessed by flow cytometry (Figures S6A–S6C). To complement these data, we further characterized the immune cell phenotypes within the tumor tissues via immunofluorescence (IF) detection. Abundant $CD45^+$ cell infiltration was observed in tumors treated with mV01 alone or in combination with PD-1 antibody, whereas these cells were virtually undetectable in tumors receiving PD-1 antibody alone or vehicle control (Figure S7A). Notably, compared to mV01 monotherapy, the combination therapy resulted in a marked increase in the expression of CD69, CD3, CD4, and CD8 markers, accompanied by greater CD45 staining intensity (Figures S7B–S7E). Collectively, these data demonstrate that combining mV01 virotherapy with mPD-1 antibody elicits broad-spectrum immune

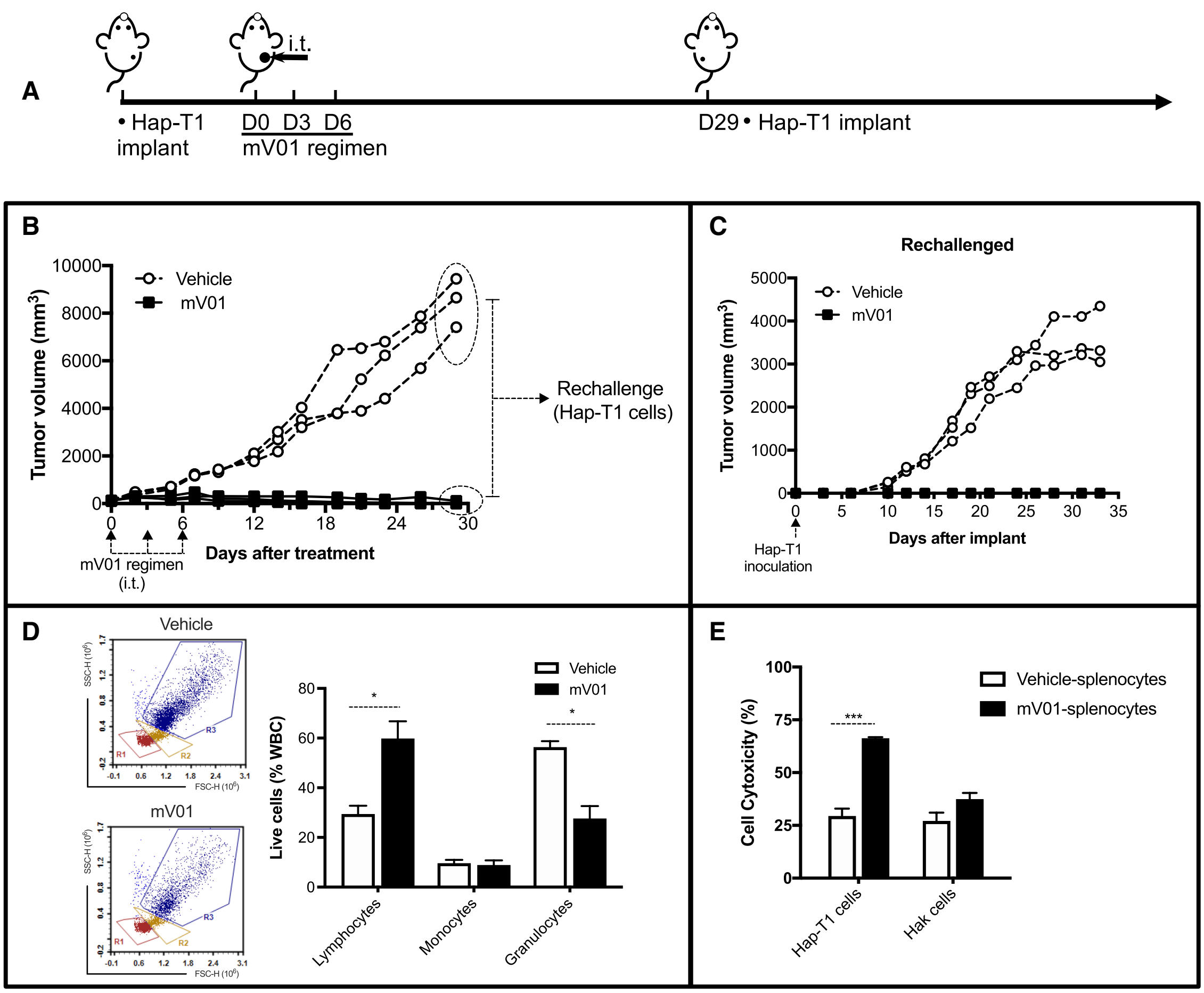

Figure 6. mV01 virotherapy eradicates tumors and induces durable anti-tumor immunity in an immunocompetent hamster model

(A) The scheme of experimental design. (B) Growth curves of primary tumors in LVG hamsters treated with mV01 or vehicle. (C) Growth curves of rechallenged tumors in LVG hamsters previously treated with vehicle or mV01. (D) Representative flow cytometry plots of peripheral blood leukocytes. Cells were first gated based on FSC-H versus SSC-H to identify lymphocytes (R1), monocytes (R2), and neutrophils (R3) (right). Quantitative analysis shows the percentage of lymphocytes, monocytes, and neutrophils relative to total WBCs. (E) *In vitro* ICD assay. Hap-T1 and Hak cells were co-cultured with splenocytes derived from vehicle- or mV01-treated Hap-T1 tumor-bearing animals. n = 3 animals per group. Values are mean ± SEM. Statistical significance was determined by an unpaired two-tailed Student's t test: *$p < 0.05$; **$p < 0.01$; ***$p < 0.001$.

activation—particularly by enhancing effector T cell responses—highlighting its potential as a synergistic anti-cancer immunotherapy strategy.

Furthermore, IF staining revealed a higher prevalence of mV01 infection in tumor sections from the combination group (>90%) compared to the mV01 monotherapy group (50%–60%) (Figure 7H). This observation was further corroborated by qPCR analysis (Figure 7I). These findings suggest that PD-1 signaling may restrict mV01 replication and i.t. spread, thereby contributing to the superior efficacy of the combination treatment.

Therapeutic potential of V01-based neoadjuvant immunotherapy

The concept of adjuvant immunotherapy has expanded to encompass oncolytic virotherapy—a highly versatile modality that remodels TME and serves as an ideal partner for combination therapies. Extending our findings beyond PD-1 antibody combination, we evaluated the combination of hV01 and IBR854, a conjugated antibody redirecting ready-to-use allogeneic NK (CAR-raNK) cell therapy, in two xenograft models: MNNG/HOS C1 human osteosarcoma xenografts and human ovarian cancer patient-derived xenografts (PDXs) in NOG mice.

In the MNNG/HOS C1 xenograft model, the combination of hV01 with IBR854 resulted in complete tumor regression, significantly outperforming either monotherapy (Figure 8A). While hV01 monotherapy exhibited inhibition of tumor growth, only the combination therapy achieved complete eradication. Similarly, in the human ovarian cancer PDX model, the combination of hV01 and IBR854 demonstrated markedly enhanced anti-tumor efficacy compared to either treatment alone (Figure 8B). These findings underscore the

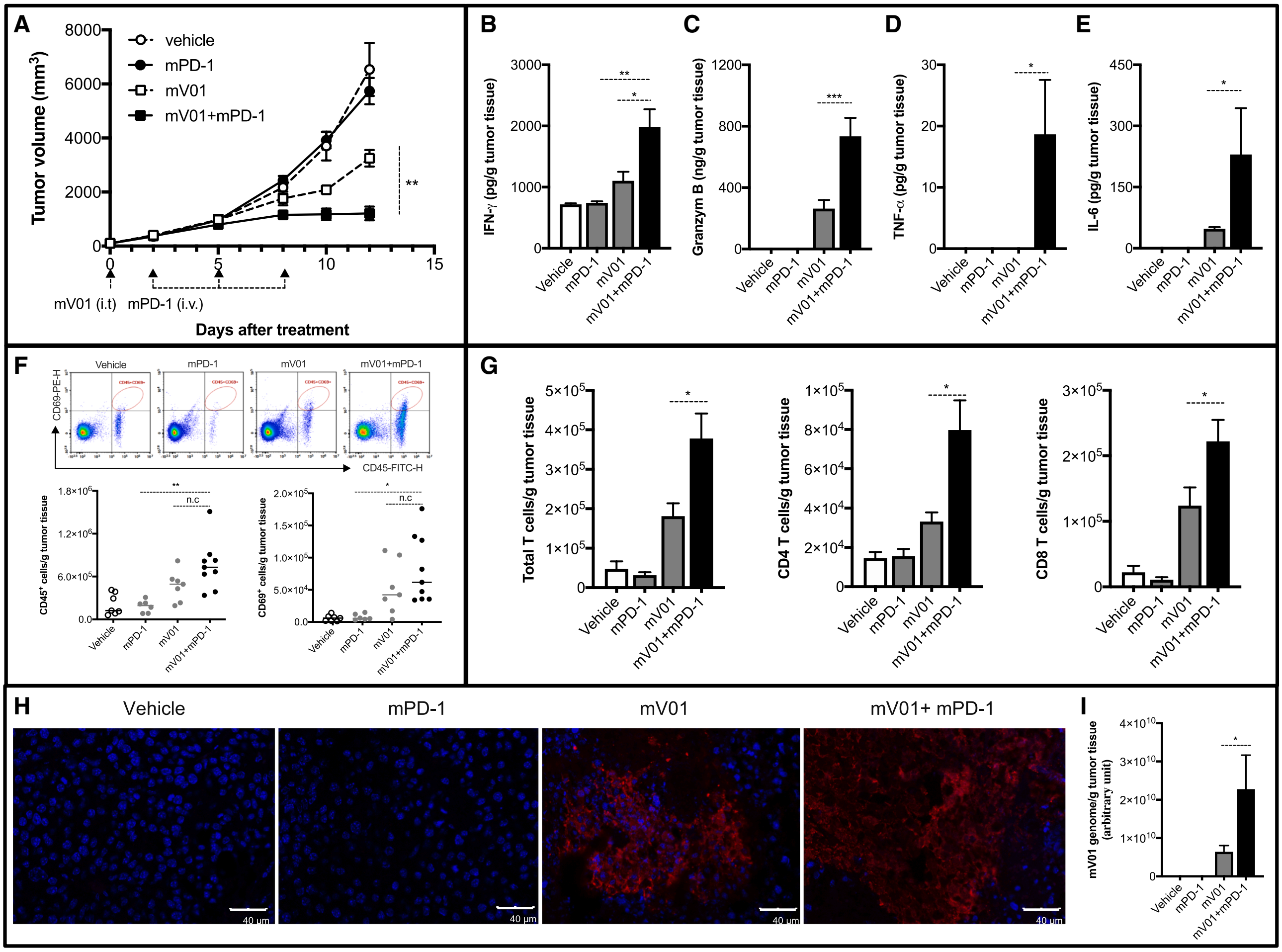

Figure 7. Blocking PD-1 signaling enhanced mV01 spread within tumors and reduced tumor burden in immunocompetent B16 tumor-bearing mice

(A) Tumor growth curves of B16 tumor-bearing mice treated with vehicle, mV01, mPD-1 antibody, or the combination of mV01 and mPD-1 antibody. (B–E) Levels of IFN-γ (B), granzyme B (C), TNF-α (D), and IL-6 (E) in tumors. (F) Representative flow cytometry plots of $CD45^+$ and $CD69^+$ staining in tumors (top) and quantitative analysis (bottom). (G) Quantitative analysis of immune cell populations, including total T cells, $CD4^+$ T cells, and $CD8^+$ T cells, in tumors. (H–I) The levels of vaccinia virus, assessed by IF staining (H) and QPCR (I), in tumor tissues after treatment. $n = 10$ mice per group. Scale bars, 40 μm. Data are presented as mean ± SEM. Statistical significance was determined by two-way ANOVA with Bonferroni post hoc correction (for tumor volumes) and by one-way ANOVA (for ELISA, QPCR and flow cytometry data): $*p < 0.05$; $**p < 0.01$; $***p < 0.001$; n.s., not significant.

therapeutic potential of combining oncolytic virotherapy with cell therapies, providing proof-of-concept therapeutic synergy. Collectively, our data indicate that hV01 serves as a potent immunomodulatory agent, providing a strong rationale for multi-modal immunotherapeutic approaches in cancer treatment.

DISCUSSION

Vaccinia virus has evolved to elicit a systemic immune response and resist clearance by complement and antibodies.[30,31] Building on these intrinsic properties, we selected the vaccinia virus Western Reserve (WR) strain and defined its tumor-targeting mechanism while optimizing safety through genetic modification. In this study, we engineered V01, an IL-21-armed recombinant oncolytic vaccinia virus, which effectively inhibited both injected and distant non-injected tumors by local administration, indicating a systemic anti-tumor effect. Furthermore, we demonstrated a synergistic anti-tumor effect through the combination of V01 virotherapy with PD-1 antibody or CAR-NK cell therapy across multiple tumor models, including syngeneic and xenograft tumor models. These results highlight V01 as a versatile platform for rational combination therapies, integrating oncolytic virotherapy with immunotherapy to enhance anti-tumor immunity and improve therapeutic outcomes.

hV01 exhibited robust replication in SKOV3 and U251 cells, as well as in liver and lung tumor tissues. Furthermore, hV01 displayed broad-spectrum oncolytic activity against multiple cancer cell lines (SKOV3, SK-HEP-1, MDA-MB-231, U251, HCT116, MCF-7, PANC-1, and A549), consistent with its *in vivo* anti-tumor efficacy. This broad

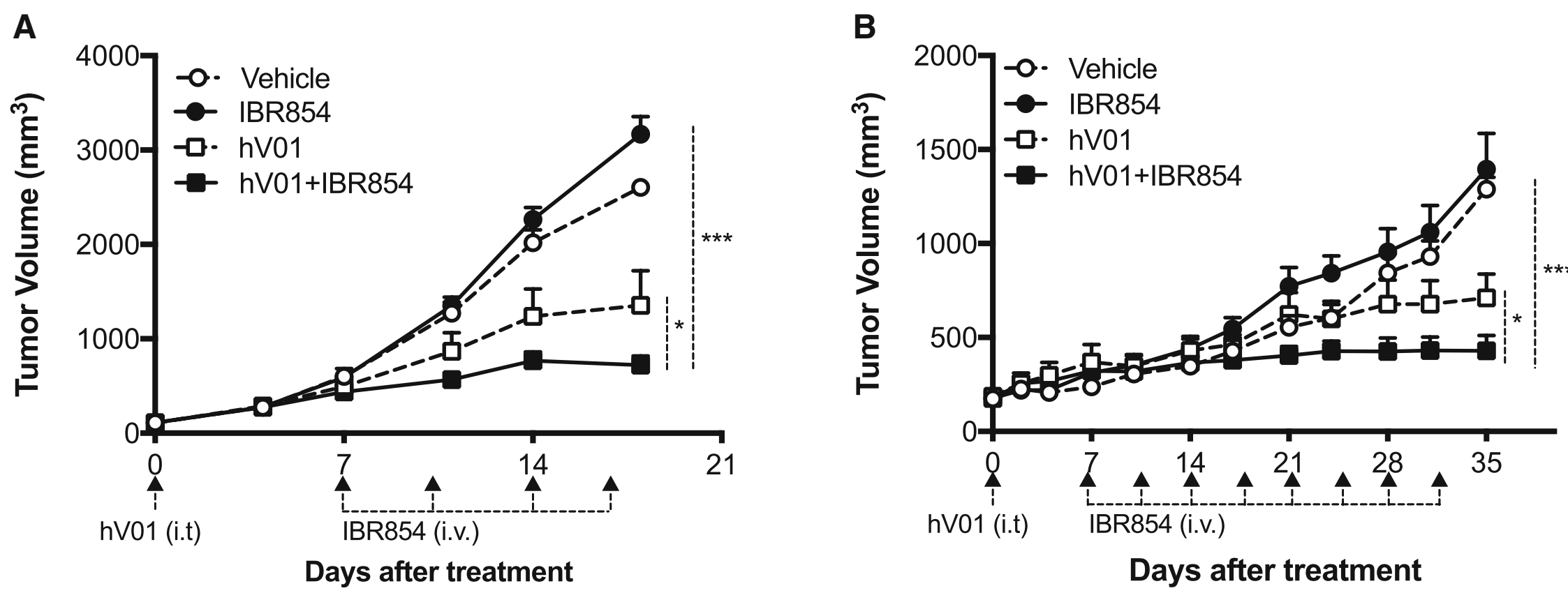

Figure 8. Combination therapy with hV01 and CAR-NK cell therapy enhanced tumor eradication across multiple tumor models

Time course of tumor growth following treatment with vehicle, hV01, IBR854, or the combination of hV01 and IBR854 in (A) MNNG/HOS C1 tumor-bearing mice and (B) human ovarian cancer PDX tumor-bearing mice. n = 8–10 mice per group. Data are presented as mean ± SEM. Statistical significance was determined by two-way ANOVA with Bonferroni correction: *$p < 0.05$; ***$p < 0.001$.

tropism may be attributed to V01's ability to enter cells independently of specific receptors, as viral fusion with the tumor cell membrane bypasses conventional receptor-mediated internalization.

Incorporating IL-21 enhances immune regulation by delivering a costimulatory signal to lymphocytes, promoting T cell activation, NK cell expansion, and the reversal of NK cell exhaustion.[15,18,19] Consistent with this, *in vitro* studies demonstrated that hV01 treatment induced superior NK cell- and PBMC-mediated cytotoxicity compared to the mock virus, with full restoration observed upon the addition of rhIL-21. Interestingly, while cytotoxicity levels were similar between the hV01 and mock + rhIL-21 groups, hV01 triggered significantly higher granzyme B production. This disparity suggests that continuous IL-21 signaling drives active granzyme B replenishment in effector cells, potentially leading to a more robust and sustained anti-tumor response, as confirmed *in vivo*. Aligning with these observations, *in vivo* rescue experiments involving the addition of rmIL-21 to mock virus treatment significantly inhibited tumor growth to a degree comparable to the mV01 group. Despite increased HMGB1 levels, neither the frequencies of MHC-II$^+$ DCs and CD4$^+$ T cells, nor the IFN-γ expression in CD4$^+$ T cells, differed between mV01- and mock-treated groups. This suggests the DC-MHCII-CD4 axis remains quiescent in the IL-21-rich TME. Conversely, CD8$^+$ T cell IFN-γ production was significantly increased, indicating that IL-21-mediated immunity selectively augments cytotoxic T cell responses. This apparent discrepancy may be driven by two mechanisms: (1) cross-presentation bias, wherein mV01-induced antigen release and DC maturation are preferentially channeled through the MHC-I pathway during early tumor immunity; and (2) IL-21 receptor signaling bias, as IL-21 directly drives CD8$^+$ T cell proliferation, terminal differentiation, and effector function,[32] while its effects on CD4$^+$ T cells are more context-dependent. Finally, although IL-21 strongly activates NK cells *in vitro* (Figures 1C and 1E; Figures S2A and S2C), its *in vivo* effect on NK-cell frequencies appears minimal (Figures S3A and S6A). This may result from transient NK activation followed by migration or apoptosis, or from IL-21 primarily enhancing NK cell function rather than proliferation.[33,34] Future studies tracking NK-cell trafficking could help elucidate these dynamics.

Our findings demonstrate that local V01 administration leads to distant tumor suppression, highlighting its potential for treating metastatic tumors. Following the V01 regimen, an increased presence of immune effector T cells was observed in both injected and non-injected tumors, indicating the activation of a systemic immune response. Notably, while IL-21 expression was significantly elevated in injected tumors, it remained undetectable in non-injected ones. This suggests that locally administered V01 may not sufficiently enter the circulation to directly reach distant sites. Interestingly, immune effector T cell levels in distant tumors were substantially higher than those in injected tumors, likely contributing to the inhibition of distant lesions and underscoring the systemic anti-tumor properties of V01. The mV01-elicited immune response was further confirmed by potent anti-tumor efficacy in the EMT6/EMT6 homologous bilateral tumor model, which was absent in the EMT6/A20 heterologous bilateral setting. Additionally, splenocytes harvested from mV01-treated Hap-T1 tumor-bearing hamsters induced effective ICD in syngeneic Hap-T1 cells, but not in Hak hamster kidney cells. This specificity is likely attributable to the presence of shared antigenic epitopes in the homologous model, whereas the heterologous model lacks these conserved immunological targets.

In agreement with the previous studies,[27,35] we demonstrated that combining the V01 regimen with PD-1 antibody further promoted the infiltration and activation of i.t. T cells, resulting in synergistic anti-tumor efficacy. Interestingly, alongside the increase in TILs, PD-1 antibody enhanced i.t. viral distribution and load, suggesting a dynamic interplay between immune reactivation and viral spread.

Restored T cell function following PD-1 antibody treatment may disrupt physical barriers within the TME or induce changes in vascular permeability,[9] thereby facilitating broader viral dissemination. Furthermore, the secretion of IFN-γ and granzyme B might alter the cellular architecture, rendering the TME more permissive to viral infection. However, it is crucial to emphasize that these data are correlative; future investigations are required to establish a direct causal relationship between PD-1 antibody and increased viral spread. Additionally, the robust oncolytic activity observed when combining hV01 with NK-based cell therapy in both *in vitro* (Figure 1C; Figure S2A) and *in vivo* models (Figure 8) underscore the significant therapeutic potential of this approach. Although the data clearly support enhanced efficacy, the current study lacks the detailed immune profiling needed to fully elucidate how the virus modulates NK cell function. Future studies will therefore focus on characterizing the immunological crosstalk and mechanistic underpinnings of this combination to optimize its clinical application.

To address the limitations of the current study, we propose the following future directions: (1) While diverse tumor models were used to assess broad oncolytic potential, future work should focus on a consistent model across *in vitro* and *in vivo* phases to derive unified mechanistic insights; (2) Synergistic effects between hV01 and IBR854 cell therapy were observed in tumor-bearing NOG mice. Because these mice lack a functional immune system, the synergy is likely driven by V01-derived IL-21 directly supporting IBR854 cell functionality rather than remodeling the host TME. Despite significant tumor burden reduction, further studies using fully humanized models are warranted to elucidate the underlying mechanisms; and (3) Although our study provides proof-of-principle that V01 priming is a novel combination strategy, future research should optimize treatment protocols, including regimen timing and tumor types, to maximize efficacy.

MATERIALS AND METHODS

Cells

Primary HUVECs were obtained from AllCells (Shanghai, China) and cultured according to the manufacturer's instructions. HaP-T1 cells were purchased from the European Collection of Authenticated Cell Cultures (ECACC; Salisbury, UK). EMT6, SKOV3, and U251 were obtained from the Cell Bank of the Chinese Academy of Sciences (Shanghai, China). Other cell lines, including Hak, MNNG/HOS C1, PANC1, B16, A20, CV1, and 143B, were purchased from the American Type Culture Collection (ATCC, VA, USA). All cells were maintained in their respective recommended culture media (Gibco, Shanghai, China) supplemented with 10% fetal bovine serum (FBS) (Gibco), 100 U/mL penicillin, and 100 μg/mL streptomycin, and incubated at 37°C in a humidified atmosphere containing 5% CO2. hPBMCs and hNK cells were isolated from the blood of healthy volunteers with informed consent, as previously described.[36,37]

Drugs

IBR854 cells were manufactured and provided by ImBioRay Inc. (Hangzhou, China). mPD-1 antibody was purchased from BioXcell (Lebanon, NH, USA). rhIL-21 protein and rmIL-21 protein were purchased from R&D Systems (Shanghai, China).

Animals

8 weeks old female C57BL/6N mice, 8 weeks old female BALB/c mice, 6 weeks old female NOD.Cg-Prkdc^{scid} Il2rg^{tm1Sug}/JicCrl mice (NOG), and LVG (SYR) hamsters were purchased from Charles River Laboratories (Beijing, China). All animals were housed under a 12 h light/12 h dark cycle (lights on at 07:00, lights off at 19:00) with *ad libitum* access to food and water. All animals were habituated for at least 3 days prior to experimental use and animal experiments were conducted under the approval of the Animal Care and Use Committee of Hangzhou Converd Inc. (ACU-21-02).

Virus generation and titration

Recombinant oncolytic viruses hV01 (expressing human IL-21) and mV01 (expressing murine IL-21) were generated using a homologous recombination system in CV1 cells. The full-length coding sequences of human or mouse IL-21 were inserted into the *Bgl*II/*Spe*I-digested donor plasmid pCB (a gift from the Zhejiang Academy of Medical Sciences, China), yielding the constructs pCB-hIL-21 and pCB-mIL-21. CV1 cells were infected with the VGF-deficient vaccinia virus strain (VSC)20 (a gift from the University of Southern California, USA) and subsequently transfected with pCB-hIL-21 or pCB-mIL-21 donor plasmids. Recombinant viruses were isolated via plaque purification and confirmed by single-clone screening. A control mock virus lacking the *IL-21* insert was generated by transfection with pCB plasmid. Viral titers were determined by plaque assay on 143B cells following viral propagation.[38]

Virus replication and production assay

Viral replication kinetics were evaluated using plaque assays. Briefly, HUVECs, U251, and SKOV3 cells were seeded in 6-well plates and infected with hV01 or the mock control virus at MOI of 0.01. Cells and culture supernatants were collected at 48 hpi, and viral titers were quantified by plaque assay.

To assess the replication competence of V01 in tumor versus normal tissues, *ex vivo* organ cultures were established. Lung and liver tissues containing tumors were harvested from tumor-bearing C57BL/6N mice, while normal lung and liver tissues were obtained from naive C57BL/6N mice. Tissue fragments were cultured *in vitro* and infected with mV01 (1×10^2 PFU per well). At 48 hpi, tissues and supernatants were harvested, and viral production was quantified by plaque assay.

Cell cytotoxicity

To evaluate hV01-induced ICD, PANC1 cells were seeded at 5×10^4 cells/well in 24-well plates and infected with hV01 or a mock control virus at MOI of 0.3. At 2 hpi, the culture medium was replaced with fresh complete medium containing effector cells at an effector-to-target (E:T) ratio of 10:1 for PBMCs and 1:1 for NK cells. The experimental groups included: hNK cells, hNK cells pre-activated with rhIL-21, hPBMCs, and hPBMCs pre-activated with rhIL-21.

Following 48 h of co-culture, cells were harvested and stained with Fixable Viability Dye eFluor 780 (Thermo Fisher Scientific, Shanghai, China). Cell viability was quantified using a NovoCyte flow cytometer (Agilent Technologies, Shanghai, China).

To evaluate hV01-induced ICD relative to antigen recognition, Hap-T1 cells and HAK cells were seeded at 5×10^4 cells/well in 24-well plates. Splenocytes were isolated from hV01-cured animals and co-cultured with the target cells at an E:T ratio of 10:1. Following a 48 h co-culture period, cells were harvested and stained with Fixable Viability Dye eFluor 780 (Thermo Fisher Scientific). Cell viability was subsequently quantified using a NovoCyte flow cytometer (Agilent Technologies).

Animal studies

IL-21 mediated anti-tumor efficacy

To establish a syngeneic tumor model, C57BL/6N mice were subcutaneously (s.c.) implanted with 2×10^5 B16 cells in the right flank. When tumors reached an average volume of approximately 100 mm^3, mice were randomized into treatment groups. The tumor received i.t. injections of mock virus (1×10^6 PFU per dose), mock virus with rmIL-21 (0.5 mg/kg), mV01 (1×10^6 PFU per dose) on days 0, 2, and 4, a schedule optimized based on our prior studies (data no shown). Tumor dimensions were measured every 2–3 days using digital calipers, and tumor volumes were calculated using the formula: volume = $[L \times (W)^2]/2$, where L represents the longest diameter and W represents the shortest perpendicular diameter. Tumor samples were harvested at day 9 after the first mV01 administration for flow cytometry and ELISA, while TDLNs were collected at days 7 and 11 for flow cytometry analysis.

Syngeneic bilateral tumor model (abscopal effect)

To establish a syngeneic bilateral tumor model, C57BL/6N mice were s.c. implanted with 2×10^5 B16 cells in both the right and left flanks. When tumors reached an average volume of approximately 100 mm^3, mice were randomized into treatment groups. The right-flank tumor received i.t. injections of mV01 (1×10^6 PFU per dose) on days 0, 2, and 4. The left-flank tumor served as an untreated distal control to evaluate abscopal effects. Tumor volumes were measured and calculated as previously described. Tumor samples were harvested at day 9 after the first mV01 administration for flow cytometry and ELISA.

Tumor antigen priming and recognition (rechallenge models)

To assess tumor antigen-specific priming and recognition, we performed homologous and heterologous tumor rechallenge experiments in BALB/c mice. Mice were initially s.c. implanted with 2×10^5 EMT6 cells in the right flank. Upon reaching a tumor volume of approximately 100 mm^3, mice received three i.t. injections of mV01 (1×10^7 PFU per dose) on days 0, 3, and 6, based on optimization from our prior studies (data no shown). Subsequently, on day 7, mice were rechallenged by s.c. implantation of either 2×10^5 EMT6 cells (homologous tumor) or 5×10^5 A20 cells (heterologous tumor) into the left flank. Tumor growth on both flanks was monitored and calculated as previously described. While standard humane endpoints typically dictate euthanasia at a tumor volume of 2,000 mm^3 for mice, our study required the assessment of re-challenged tumors, which manifest as large-volume masses of primary implanted tumors in the late stage. Therefore, under the approval of the ACU-21-02, animals were permitted to carry tumors up to 8,000 mm^3 provided that they maintained a body condition score (BCS) of $\geq$2.5 and showed no signs of distress, ulceration, or impairment of normal bodily functions. Animals were euthanized immediately if any signs of morbidity were observed, regardless of tumor size. Tumor samples were harvested at day 20 after the first mV01 administration for flow cytometry.

Immunological memory evaluation

To evaluate the induction of immunological memory, we conducted a rechallenge study in LVG hamsters. Animals were s.c. implanted with 2×10^6 Hap-T1 cells in the right flank. When tumors reached an average volume of approximately 150 mm^3, animals received three i.t. injections of mV01 (1×10^7 PFU per dose) on days 0, 3, and 6 our prior studies (data no shown). On day 29, animals that achieved a complete response following mV01 treatment, along with vehicle-treated control animals, were rechallenged with s.c. implantation of 2×10^6 Hap-T1 cells in the left flank. Tumor volumes were measured and calculated as previously described. While standard humane endpoints typically dictate euthanasia at a tumor volume of 3,000 mm^3 for hamster, our study required the assessment of re-challenged tumors, which manifest as large-volume masses of primary tumors in the late stage. Therefore, under the approval of the ACU-21-02, animals were permitted to carry tumors up to 8,000 mm^3 provided that they maintained a BCS of $\geq$2.5 and showed no signs of distress, ulceration, or impairment of normal bodily functions. Animals were euthanized immediately if any signs of morbidity were observed, regardless of tumor size. Peripheral blood was collected via orbital vein puncture and spleen tissues were harvested at day 60 after the first mV01 administration for flow cytometry analysis and PBMC isolation.

Combination efficacy studies

1) PD-1 antibody combination: syngeneic C57BL/6N mice were s.c. inoculated with 2×10^5 B16 cells in the right flank. Treatment consisted of a single i.t. injection of mV01 (3×10^4 PFU) on day 0, followed by i.v. administration of mPD-1 antibody (5 mg/kg) on days 2, 5, and 8. Tumor volumes and body weight were measured throughout the study and calculated as previously described. Tumor samples were harvested at day 12 after the first mV01 administration for flow cytometry, ELISA, IF, and qPCR detections.
2) IBR854 cell therapy combination: NOG mice were s.c. implanted with either 1×10^7 MNNG/HOS C1 cells or human ovarian cancer PDX in the right flank. Mice received a single i.t. injection of hV01 (1×10^3 PFU) on day 0, followed by i.v. administration of IBR854 cells (5×10^6 cells) starting on day 7. IBR854 cells were administered twice weekly until the end of the study. Tumor volumes were measured and calculated as previously described.

ELISA assay

The levels of granzyme B, IFN-γ, TNF-α, IL-6, and IL-21 were quantified using ELISA kits according to the manufacturers' protocols. Absorbance was measured using a VICTOR Nivo Multimode Plate Reader (PerkinElmer, Shanghai, China), and data acquisition and analysis were performed using VICTOR Nivo control software v.2.0.2 (PerkinElmer). The following ELISA kits were used: Mouse IFN-γ, Mouse IL-6, Mouse Granzyme B, Mouse IL-21, and Human Granzyme B (R&D Systems); Mouse TNF-α High Sensitivity (Absin Bioscience, Shanghai, China); Hamster IFN-γ (FineTest, Wuhan, China) and Mouse HMGB1 ELISA Kit (FineTest).

qPCR assay

V01 virus genomic DNA was extracted using the QIAamp MinElute Virus Spin Kit (QIAGEN, Shanghai, China) following the manufacturer's protocol. The qPCR assay was conducted using Premix Ex Taq (Takara, Dalian, China) on a real-time PCR system. The specific primer and probe were synthesized by LifeTech Ltd. (Shanghai, China) with the following sequences: forward primer: 5′-GAA CATTTTTGGCAGAGAGAGCC-3'; reverse primer: 5′-CAACTCT TAGCCGAAGCGTATGAG-3'; probe: 5′-FAM-CAGGCTACCAG TTCAA-NFQ-MGB-3'. All reactions were performed in triplicate. A no-template control was included in each run to monitor for potential contamination.

Flow cytometry-base analysis

Flow cytometry was performed on a NovoCyte flow cytometer, and data were analyzed using NovoExpress software (Agilent Technologies). The list of antibodies used and panel designations are provided in Tables S1 and S2. The gating strategy is illustrated in Figures S8–S10.

For TIL profile analysis in mice models

Tumor tissues were harvested, resected, and mechanically dissociated using a gentleMACS Octo Dissociator (Miltenyi Biotec, Shanghai, China) following enzymatic digestion with 0.5 mg/mL type 1/4 collagenase (Diamond, Shanghai, China) at 37°C for 30 min under gentle rocking. The digested tissue was filtered through a 40 μm cell strainer and washed with phosphate-buffered saline (PBS) to generate a single-cell suspension. Cells were stained with Fixable Viability Dye eFluor 780 (Thermo Fisher Scientific) to exclude dead cells. Initial gating was performed on live, singlet lymphocytes identified by forward scatter (FSC) and side scatter (SSC) parameters (using height, area, and width to exclude doublets), followed by gating on $CD45^+$ cells. Immune subsets were defined as follows: activated lymphocytes ($CD45^+CD69^+$), total T cells ($CD45^+CD3^+$), CD4 T cells ($CD3^+$ $CD4^+$), CD8 T cells ($CD3^+CD8a^+$), DCs ($DC11c^+MHCII^+$), and antigen-presenting cells (APCs) ($CD45^+CD86^+$). The list of antibodies used and panel designations are provided in Tables S1 and S2. The gating strategy is illustrated in Figures S8A, S8B, and S9.

IFN-γ intracellular staining assay

Cells were resuspended in RPMI 1640 culture medium supplemented with 5% FBS and stimulated with a cocktail containing Phorbol 12-myristate 13-acetate (PMA) (Absin Bioscience) and ionomycin at final concentrations of 50 ng/mL and 1 μg/mL, respectively. After 1 h of incubation, 1× Brefeldin A (Thermo Fisher Scientific) was added, and the cells were incubated for an additional 4 h. Cells were then harvested, fixed, permeabilized, and stained intracellularly for IFN-γ with surface staining for CD4 or CD8, followed by flow cytometric analysis. The list of antibodies used and panel designations are provided in Tables S1 and S2. The gating strategy is illustrated in Figure S8C.

For cell profile analysis in TDLNs

TDLNs were harvested from tumor-bearing mice and mechanically dissociated using a gentleMACS Octo Dissociator (Miltenyi Biotec, Shanghai, China) following enzymatic digestion with 0.5 mg/mL type 1/4 collagenase (Diamond, Shanghai, China) at 37°C for 30 min under gentle rocking. The digested tissue was filtered through a 40 μm cell strainer and washed with PBS to generate a single-cell suspension. Cells were stained with Fixable Viability Dye eFluor 780 (Thermo Fisher Scientific) to exclude dead cells. Initial gating was performed on live, singlet lymphocytes identified by FSC and SSC parameters (using height, area, and width to exclude doublets), followed by gating on $CD45^+$ cells. Immune subsets were defined as follows: DCs ($DC11c^+MHCII^+$), naive T cells ($CD44^-CD62L^+$), transmission electron microscopy (TEM) ($CD44^+CD62L^-$), and CEM ($CD44^+CD62L^+$). The list of antibodies used and panel designations are provided in Tables S1 and S2. The gating strategy is illustrated in Figure S8D.

For peripheral blood immune cell analysis in the hamster model

Blood was collected via orbital vein puncture. WBCs were isolated using two cycles of erythrocyte lysis buffer (TianGen Bioteche, Beijin, China) and subsequently suspended in RPMI-1640. Cells were stained with Fixable Viability Dye eFluor 780 (Thermo Fisher Scientific) to assess viability. Cells were first gated based on FSC height (FSC-H) versus SSC height (SSC-H) to distinguish lymphocytes, monocytes, and neutrophils.[39] Following this, cells were stained with fluorescein isothiocyanate (FITC) fluorescein-labeled anti-CD45 PE/Cy7 (Novus Biologicals) and the gating strategy is illustrated in Figure S10.

IF staining

Tumor tissues were collected, fixed in 10% formalin, embedded in paraffin, and sectioned into 5-μm-thick slices. The slices were heated in citrate buffer (10 mM sodium citrate, 0.05% Tween 20, pH 6.0) at 95°C for 20 min, then blocked with 10% goat serum for 60 min at room temperature. IF staining was performed using a rabbit polyclonal anti-vaccinia virus antibody (Abcam, Shanghai, China), followed by incubation with an Alexa Fluor 594-conjugated goat anti-rabbit IgG secondary antibody (Abcam). After washing, sections were counterstained with Hoechst 33342 (Beyotime, Hangzhou, China) for nuclear visualization and mounted with coverslips. Images were acquired using an Olympus FV3000RS confocal microscope (Olympus, Shanghai, China).

Statistics

Statistical analyses were performed, and graphs were generated using GraphPad Prism 9.0 (GraphPad Software, San Diego, CA, USA). Comparisons between two groups were conducted using an unpaired Student's *t* test, while one-way ANOVA was employed for comparisons among multiple groups. To assess the effects of two factors with repeated measurements over time (e.g., tumor growth curves), a two-way repeated-measures ANOVA with Bonferroni post hoc correction was used. Data are presented as mean ± standard error of the mean (SEM). p values <0.05 were considered statistically significant and are denoted as follows: $^{*}p < 0.05$, $^{**}p < 0.01$, and $^{***}p < 0.001$. Specific statistical tests and sample sizes (n) are detailed in each figure legend.

DATA AND CODE AVAILABILITY

All data generated during this study were stored on the internet data-room server of Hangzhou Converd Ltd. and can be accessed from the corresponding author.

ACKNOWLEDGMENTS

This work was supported by the joint funds for the Innovation of Science and Technology of Fujian Province, China (grant no. 2024Y9469). We thank Dr. Shiyi Chen (University of Southern California) for generously providing the VSC20 vaccinia virus backbone and Dr. Hui Yan (Zhejiang Academy of Medical Sciences) for supplying the pCB plasmid. We also acknowledge Ms. Xiuqin Zhang for her assistance with manuscript formatting.

AUTHOR CONTRIBUTIONS

J.F. and J.C. managed project and wrote manuscript; R.Z., T.W., K.Y., Y.Q., and C.H. performed the experiments; Y.W. and H.Z. participated experimental design.

DECLARATION OF INTERESTS

J.F., R.Z., and T.W. are inventors on patents (ZL201710882027.1; ZL201880062059.X; P7248325) submitted by Hangzhou ConVerd Co. Ltd., Hangzhou, China.

SUPPLEMENTAL INFORMATION

Supplemental information can be found online at https://doi.org/10.1016/j.omton.2026.201348.

REFERENCES

1. Wolchok, J.D., Chiarion-Sileni, V., Gonzalez, R., Rutkowski, P., Grob, J.-J., Cowey, C.L., Lao, C.D., Wagstaff, J., Schadendorf, D., Ferrucci, P.F., et al. (2017). Overall Survival with Combined Nivolumab and Ipilimumab in Advanced Melanoma. N. Engl. J. Med. *377*, 1345–1356. https://doi.org/10.1056/NEJMoa1709684.

2. Teng, M.W.L., Ngiow, S.F., Ribas, A., and Smyth, M.J. (2015). Classifying Cancers Based on T-cell Infiltration and PD-L1. Cancer Res. *75*, 2139–2145. https://doi.org/10.1158/0008-5472.CAN-15-0255.

3. Topalian, S.L., Taube, J.M., Anders, R.A., and Pardoll, D.M. (2016). Mechanism-driven biomarkers to guide immune checkpoint blockade in cancer therapy. Nat. Rev. Cancer *16*, 275–287. https://doi.org/10.1038/nrc.2016.36.

4. Morad, G., Helmink, B.A., Sharma, P., and Wargo, J.A. (2021). Hallmarks of response, resistance, and toxicity to immune checkpoint blockade. Cell *184*, 5309–5337.

5. Lawler, S.E., Speranza, M.-C., Cho, C.-F., and Chiocca, E.A. (2017). Oncolytic Viruses in Cancer Treatment: A Review. JAMA Oncol. *3*, 841–849. https://doi.org/10.1001/jamaoncol.2016.2064.

6. Pearl, T.M., Markert, J.M., Cassady, K.A., and Ghonime, M.G. (2019). Oncolytic Virus-Based Cytokine Expression to Improve Immune Activity in Brain and Solid Tumors. Mol. Ther. Oncolytics *13*, 14–21. https://doi.org/10.1016/j.omto.2019.03.001.

7. Achard, C., Surendran, A., Wedge, M.-E., Ungerechts, G., Bell, J., and Ilkow, C.S. (2018). Lighting a Fire in the Tumor Microenvironment Using Oncolytic Immunotherapy. EBioMedicine *31*, 17–24. https://doi.org/10.1016/j.ebiom.2018.04.020.

8. McCart, J.A., Puhlmann, M., Lee, J., Hu, Y., Libutti, S.K., Alexander, H.R., and Bartlett, D.L. (2000). Complex interactions between the replicating oncolytic effect and the enzyme/prodrug effect of vaccinia-mediated tumor regression. Gene Ther. *7*, 1217–1223. https://doi.org/10.1038/sj.gt.3301237.

9. Thorne, S.H., Hwang, T.-H.H., O'Gorman, W.E., Bartlett, D.L., Sei, S., Kanji, F., Brown, C., Werier, J., Cho, J.-H., Lee, D.-E., et al. (2007). Rational strain selection and engineering creates a broad-spectrum, systemically effective oncolytic poxvirus, JX-963. J. Clin. Investig. *117*, 3350–3358. https://doi.org/10.1172/JCI32727.

10. Buller, R.M., Chakrabarti, S., Cooper, J.A., Twardzik, D.R., and Moss, B. (1988). Deletion of the vaccinia virus growth factor gene reduces virus virulence. J. Virol. *62*, 866–874. https://doi.org/10.1128/JVI.62.3.866-874.1988.

11. Martuza, R.L., Malick, A., Markert, J.M., Ruffner, K.L., and Coen, D.M. (1991). Experimental Therapy of Human Glioma by Means of a Genetically Engineered Virus Mutant. Science *252*, 854–856. https://doi.org/10.1126/science.1851332.

12. Liu, T.-C., Galanis, E., and Kirn, D. (2007). Clinical trial results with oncolytic virotherapy: a century of promise, a decade of progress. Nat. Rev. Clin. Oncol. *4*, 101–117. https://doi.org/10.1038/ncponc0736.

13. Chaurasiya, S., and Warner, S. (2017). Viroimmunotherapy for Colorectal Cancer: Clinical Studies. Biomedicines *5*, 11. https://doi.org/10.3390/biomedicines5010011.

14. Russell, L., and Peng, K.-W. (2018). The emerging role of oncolytic virus therapy against cancer. Chin. Clin. Oncol. *7*, 16. https://doi.org/10.21037/cco.2018.04.04.

15. Leonard, W.J., and Spolski, R. (2005). Interleukin-21: a modulator of lymphoid proliferation, apoptosis and differentiation. Nat. Rev. Immunol. *5*, 688–698. https://doi.org/10.1038/nri1688.

16. Moroz, A., Eppolito, C., Li, Q., Tao, J., Clegg, C.H., and Shrikant, P.A. (2004). IL-21 Enhances and Sustains CD8+ T Cell Responses to Achieve Durable Tumor Immunity: Comparative Evaluation of IL-2, IL-15, and IL-211. J. Immunol. *173*, 900–909. https://doi.org/10.4049/jimmunol.173.2.900.

17. Allard, E.-L., Hardy, M.-P., Leignadier, J., Marquis, M., Rooney, J., Lehoux, D., and Labrecque, N. (2007). Overexpression of IL-21 promotes massive CD8+ memory T cell accumulation. Eur. J. Immunol. *37*, 3069–3077. https://doi.org/10.1002/eji.200637017.

18. Parrish-Novak, J., Dillon, S.R., Nelson, A., Hammond, A., Sprecher, C., Gross, J.A., Johnston, J., Madden, K., Xu, W., West, J., et al. (2000). Interleukin 21 and its receptor are involved in NK cell expansion and regulation of lymphocyte function. Nature *408*, 57–63. https://doi.org/10.1038/35040504.

19. Seo, H., Jeon, I., Kim, B.-S., Park, M., Bae, E.-A., Song, B., Koh, C.-H., Shin, K.-S., Kim, I.-K., Choi, K., et al. (2017). IL-21-mediated reversal of NK cell exhaustion facilitates anti-tumour immunity in MHC class I-deficient tumours. Nat. Commun. *8*, 15776. https://doi.org/10.1038/ncomms15776.

20. Shanley, M., Daher, M., Dou, J., Li, S., Basar, R., Rafei, H., Dede, M., Gumin, J., Pantaleón García, J., Nunez Cortes, A.K., et al. (2024). Interleukin-21 engineering enhances NK cell activity against glioblastoma via CEBPD. Cancer Cell *42*, 1450–1466.e11. https://doi.org/10.1016/j.ccell.2024.07.007.

21. Søndergaard, H., Galsgaard, E.D., Bartholomaeussen, M., Straten, P.T., Ødum, N., and Skak, K. (2010). Intratumoral Interleukin-21 Increases Antitumor Immunity, Tumor-infiltrating CD8+ T-cell Density and Activity, and Enlarges Draining Lymph Nodes. J. Immunother. *33*, 236–249. https://doi.org/10.1097/CJI.0b013e3181c0c1cb.

22. Sivakumar, P.V., Garcia, R., Waggie, K.S., Anderson-Haley, M., Nelson, A., and Hughes, S.D. (2013). Comparison of vascular leak syndrome in mice treated with IL21 or IL2. Comp. Med. *63*, 13–21.

23. Petrella, T.M., Tozer, R., Belanger, K., Savage, K.J., Wong, R., Smylie, M., Kamel-Reid, S., Tron, V., Chen, B.E., Hunder, N.N., et al. (2012). Interleukin-21 has activity in patients with metastatic melanoma: a phase II study. J. Clin. Oncol. *30*, 3396–3401. https://doi.org/10.1200/JCO.2011.40.0655.

24. Bhatia, S., Curti, B., Ernstoff, M.S., Gordon, M., Heath, E.I., Miller, W.H., Puzanov, I., Quinn, D.I., Flaig, T.W., VanVeldhuizen, P., et al. (2014). Recombinant

interleukin-21 plus sorafenib for metastatic renal cell carcinoma: a phase 1/2 study. J. Immunother. Cancer *2*, 2. https://doi.org/10.1186/2051-1426-2-2.

25. Guo, Z.S., Lu, B., Guo, Z., Giehl, E., Feist, M., Dai, E., Liu, W., Storkus, W.J., He, Y., Liu, Z., and Bartlett, D.L. (2019). Vaccinia virus-mediated cancer immunotherapy: cancer vaccines and oncolytics. J. Immunother. Cancer *7*, 6. https://doi.org/10.1186/s40425-018-0495-7.

26. Kaufman, H.L., Kohlhapp, F.J., and Zloza, A. (2015). Oncolytic viruses: a new class of immunotherapy drugs. Nat. Rev. Drug Discov. *14*, 642–662.

27. Liu, Z., Ravindranathan, R., Kalinski, P., Guo, Z.S., and Bartlett, D.L. (2017). Rational combination of oncolytic vaccinia virus and PD-L1 blockade works synergistically to enhance therapeutic efficacy. Nat. Commun. *8*, 14754. https://doi.org/10.1038/ncomms14754.

28. Bommareddy, P.K., Shettigar, M., and Kaufman, H.L. (2018). Integrating oncolytic viruses in combination cancer immunotherapy. Nat. Rev. Immunol. *18*, 498–513. https://doi.org/10.1038/s41577-018-0014-6.

29. Burchett, R., Walsh, S., Wan, Y., and Bramson, J.L. (2020). A rational relationship: Oncolytic virus vaccines as functional partners for adoptive T cell therapy. Cytokine Growth Factor Rev. *56*, 149–159. https://doi.org/10.1016/j.cytogfr.2020.07.003.

30. Buller, R.M., and Palumbo, G.J. (1991). Poxvirus pathogenesis. Microbiol. Rev. *55*, 80–122. https://doi.org/10.1128/mr.55.1.80-122.1991.

31. Smith, G.L., Symons, J.A., Khanna, A., Vanderplasschen, A., and Alcami, A. (1997). Vaccinia virus immune evasion. Immunol. Rev. *159*, 137–154. https://doi.org/10.1111/j.1600-065x.1997.tb01012.x.

32. Li, X.-Y., Xie, X.-Q., Wei, B.-C., Sun, X.-Z., Chen, M.-X., Liu, R.-F., Tao, Q.-X., Huang, Y.-H., Wang, Q., Ma, S.-S., et al. (2026). IL-21 enhances the cytotoxicity of intratumoral CD8+ T cells, improving radiation efficacy. JCI Insight *11*, e190531. https://doi.org/10.1172/jci.insight.190531.

33. Brady, J., Carotta, S., Thong, R.P.L., Chan, C.J., Hayakawa, Y., Smyth, M.J., and Nutt, S.L. (2010). The Interactions of Multiple Cytokines Control NK Cell Maturation. J. Immunol. *185*, 6679–6688. https://doi.org/10.4049/jimmunol.0903354.

34. Wendt, K., Wilk, E., Buyny, S., Schmidt, R.E., and Jacobs, R. (2007). Interleukin-21 differentially affects human natural killer cell subsets. Immunology *122*, 486–495. https://doi.org/10.1111/j.1365-2567.2007.02675.x.

35. Zamarin, D., Ricca, J.M., Sadekova, S., Oseledchyk, A., Yu, Y., Blumenschein, W.M., Wong, J., Gigoux, M., Merghoub, T., and Wolchok, J.D. (2018). PD-L1 in tumor microenvironment mediates resistance to oncolytic immunotherapy. J. Clin. Investig. *128*, 5184. https://doi.org/10.1172/JCI125039.

36. Grievink, H.W., Luisman, T., Kluft, C., Moerland, M., and Malone, K.E. (2016). Comparison of Three Isolation Techniques for Human Peripheral Blood Mononuclear Cells: Cell Recovery and Viability, Population Composition, and Cell Functionality. Biopreserv. Biobanking *14*, 410–415. https://doi.org/10.1089/bio.2015.0104.

37. Phan, M.-T.T., Lee, S.-H., Kim, S.-K., and Cho, D. (2016). Expansion of NK Cells Using Genetically Engineered K562 Feeder Cells. In Natural Killer Cells: Methods and Protocols, S.S. Somanchi, ed. (Springer), pp. 167–174.

38. Ausubel, F.M. (1999). Short Protocols in Molecular Biology : A Compendium of Methods from Current Protocols in Molecular Biology, 4th ed. (Wiley).

39. Hendrickx, S., Bulté, D., Van den Kerkhof, M., Cos, P., Delputte, P., Maes, L., and Caljon, G. (2019). Immunosuppression of Syrian golden hamsters accelerates relapse but not the emergence of resistance in *Leishmania infantum* following recurrent miltefosine pressure. Int. J. Parasitol.: Drugs Drug Resist. *9*, 1–7. https://doi.org/10.1016/j.ijpddr.2018.12.001.

OMTON, Volume *34*

Supplemental information

Interleukin-21 expressing oncolytic vaccinia virus elicits systemic anti-tumor immune response and synergizes with immunotherapies

Rong Zhang, Tingting Wang, Yu Wang, Chenggang Hong, Yun Qin, Kangchen Ye, Hu Zhao, Fang Hu, Jinlu Cai, and Jin Fu

Supplemental Methods

Cells. A375, MDA-MB-231, and U251 were obtained from the Cell Bank of the Chinese Academy of Sciences. Other cell lines, including MNNG/HOS C1, HCT-116, MCF-7, PANC1 and SKOV3 were purchased from the American Type Culture Collection. All cells, excluding MDA-MB-231 cells, were maintained in their respective recommended culture media supplemented with 10% FBS, 100 U/mL penicillin, and 100 μg/mL streptomycin, and incubated at 37°C in a humidified atmosphere containing 5% CO2. MDA-MB-231 cells were cultured at 37°C in the absent of CO2.

Cell cytotoxicity. To assess the oncolytic activity of hV01, we performed a dose-response analysis using eight human cancer cell lines: MNNG/HOS C1, U251, SKOV3, A375, MCF-7, PANC-1, MDA-MB-231, and HCT-116. Cells were seeded at a density of 5,000 cells per well in 96-well plates and infected with serial dilutions of hV01. After 48 hpi, cell viability was quantified using the MTT assay (Invitrogen, Shanghai, China) according to the manufacturer's instructions. Absorbance was measured at 490 nm using a VICTOR Nivo™ Multimode Plate Reader (PerkinElmer). Oncolytic efficacy was expressed as the percentage of cell viability relative to mock-infected controls. Dose-response curves were generated to determine the half-maximal inhibitory concentration (IC_{50}) of hV01.

To evaluate hV01-induced ICD, MDA-MB-231 cells were seeded at 5×10^4 cells/well in 24-well plates and infected with hV01 or a mock control virus at MOI of 1.0. After 2 hpi, the culture medium was replaced with fresh complete medium containing effector cells at an E:T ratio of 5:1 for hPBMCs and 0.5:1 for hNK cells. The effector groups included: hNK cells, hNK cells pre-activated with hrIL-21, hPBMCs, or hPBMCs pre-activated with hrIL-21. Following 48 hours of co-culture, cells were harvested and stained with Fixable Viability Dye eFluor™ 780

(ThermoFisher Scientific). Cell viability was quantified using a NovoCyte flow cytometer (Agilent Technologies, Shanghai, China).

Animal studies. Syngeneic C57BL/6N mice were s.c. inoculated with 5×10^5 MC38 cells in the right flank. Treatment consisted of a single i.t. injection of mV01 (3×10^4 PFU) on day 0, followed by i.v. administration of mPD-1 antibody (5 mg/kg) on days 2, 5, and 8. Tumor volumes and body weight were measured throughout the study and calculated as previously described.

Flow cytometry-base immune cell analysis.

Tumor samples were prepared as previously described, and cells were subsequently stained with Fixable Viability Dye eFluor™ 780 (ThermoFisher Scientific). Initial gating was performed on live, singlet lymphocytes identified by forward scatter (FSC) and side scatter (SSC) parameters (using height, area, and width to exclude doublets), followed by gating on $CD45^+$ cells. Immune subsets were defined as follows: NK cells ($CD45^+NK1.1^+$), macrophages ($CD45^+F4/80^+$) and B cells ($CD45^+CD3^-CD19^+$). The list of antibodies and panel designations are provided in Tables S1 and S2.

Immunohistology.

IF staining: Tumor tissues slices were heated in Citrate Buffer (10 mM sodium citrate, 0.05% Tween-20, pH 6.0) at 95°C for 20 minutes, then blocked with 10% goat serum for 60 mins at room temperature. IF staining was performed using primary antibodies, followed by incubation with fluorescence-conjugated secondary antibodies. The antibody sets were following: for CD45 detection, Primary: Rabbit monoclonal anti-CD45 [EPR20033] (Abcam), Secondary: Goat Anti-Rabbit IgG H&L Alexa Fluor® 488 (Abcam); for CD69/CD3ε detection, Primary: Rabbit polyclonal anti-CD69 (Abcam) and Mouse monoclonal anti-CD3ε [EPR22667-12] (Abcam),

Secondary: Goat Anti-Rabbit IgG H&L Alexa Fluor® 594 (Abcam); for CD4/CD8 detection, Primary: Rat monoclonal anti-CD4 [GK1.5] (R&D Systems) and Rat monoclonal anti-CD8 [53-6.7] (Novus), Secondary: Goat Anti-Rat IgG H&L Alexa Fluor® 488 (Abcam). After washing, sections were counterstained with Hoechst 33342 (Beyotime) and mounted with coverslips. Images were acquired using an Olympus FV3000RS confocal microscope (Olympus).

H&E staining: At the end of experiment, liver and kidney tissues were harvested, fixed in 10% formalin, embedded in paraffin, and sectioned into 5-μm-thick slices. The tissue sections were deparaffinized in xylene and rehydrated through a graded series of ethanol solutions. The sections were then stained with hematoxylin and eosin using HE Staining System (KohyPath, Shanghai, China) according to the manufacturers' protocol. The slides were dehydrated, cleared, and mounted with coverslips. Images were acquired using an Olympus FV3000RS confocal microscope (Olympus)

Figures

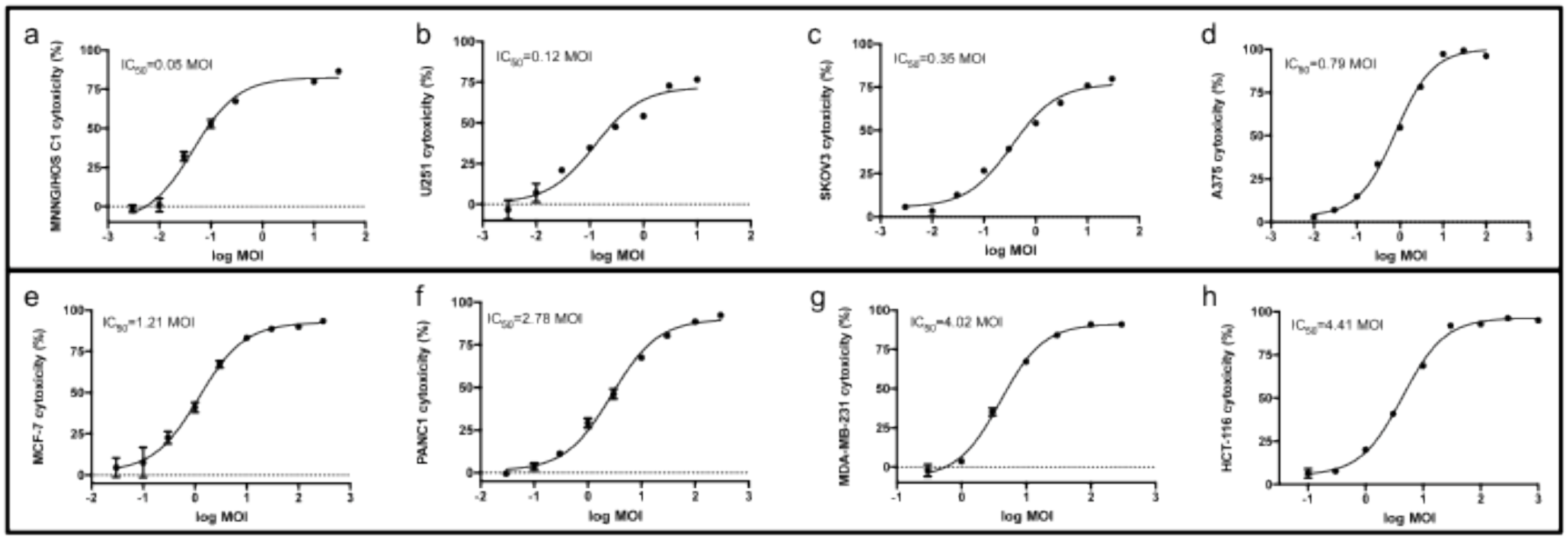

Figure S1. hV01 effectively inhibited the growth of various tumor cells. (a-h) Dose-dependent effects of hV01 on the cell viability of MNNG/HOS C1 (a), U251 (b), SKOV3 (c), A375 (d), MCF-7 (e), PANC1 (f), MDA-MB-231 (g), and HCT116 (h) cells after 48 hpi. Values were the means of three independent experiments, and data are shown as means ± SEM.

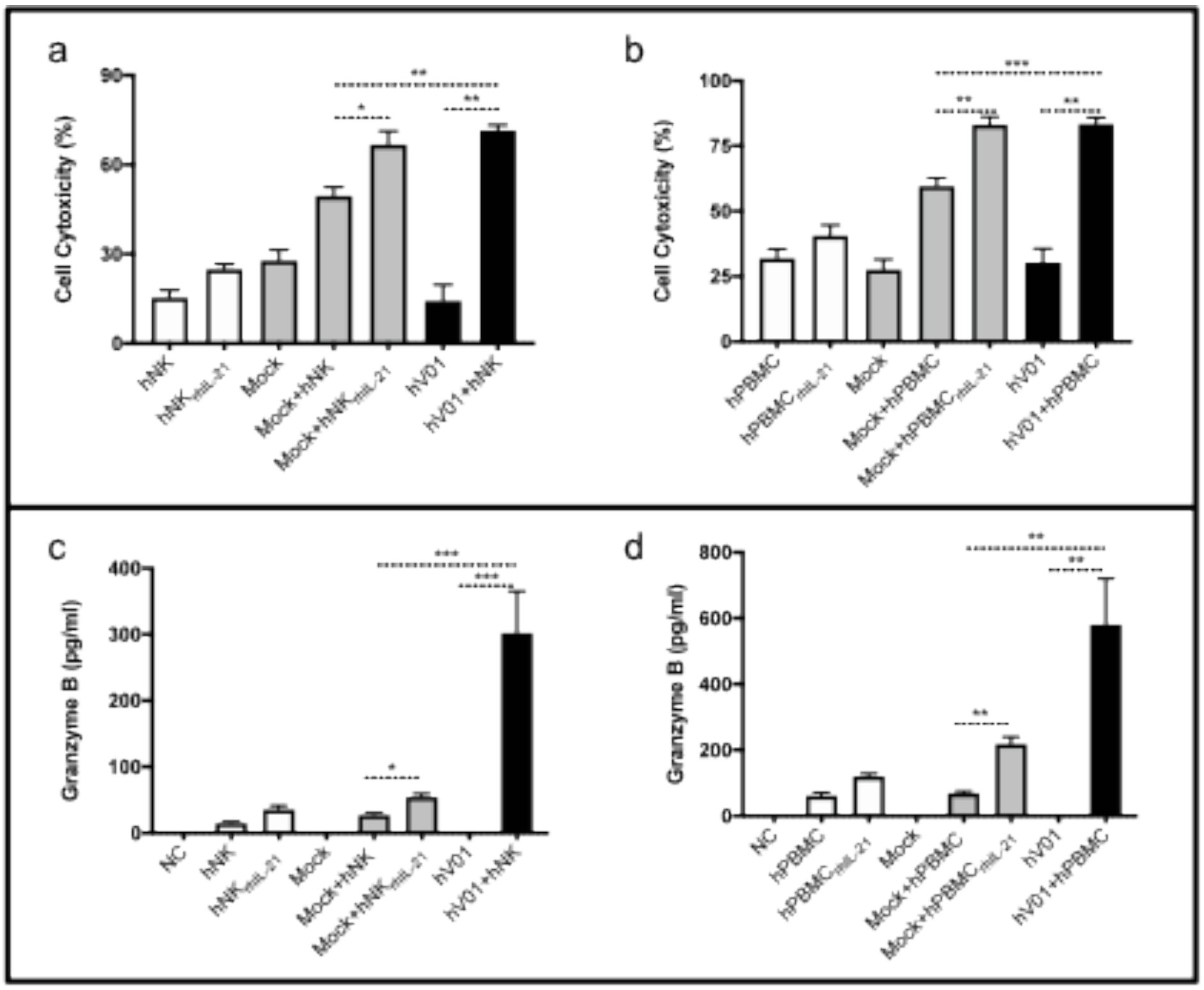

Figure S2. hIL-21 enhanced ICD in vitro. (a, b) Cell cytotoxicity and (c, d) granzyme B production were measured in MDA-MB-231 cells infected with hV01 or mock virus (MOI = 1.0) and co-cultured with hNK cells (a, c) or hPBMCs (b, d). Data represent the mean ± SEM of three independent experiments. Statistical significance was determined by one-way ANOVA: * $P < 0.05$; ** $P < 0.01$; *** $P < 0.001$.

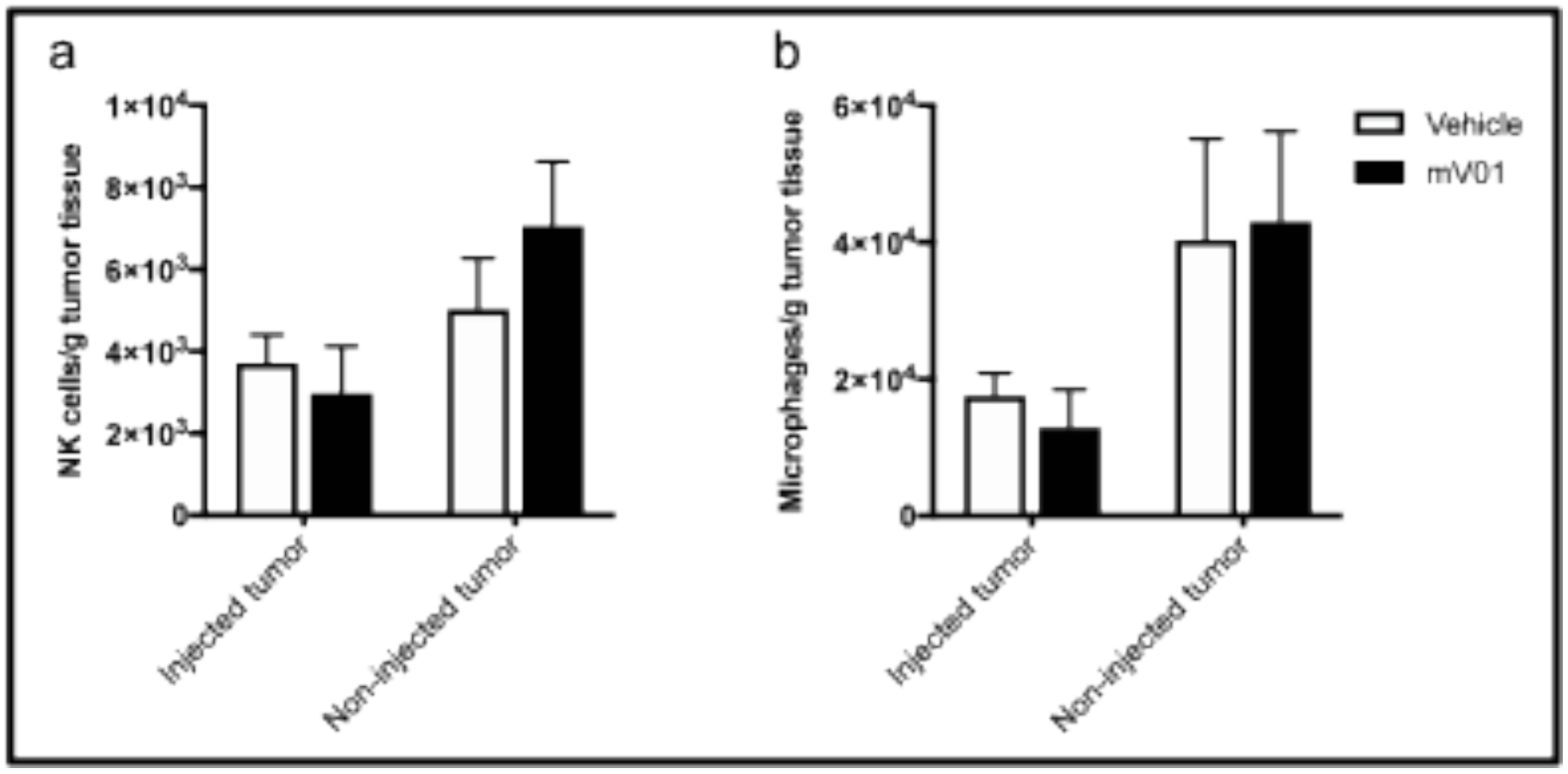

Figure S3. Quantitative analysis of immune cell populations of NK cell (a) and Microphage (b) on the injected tumors and non-injected tumors of B16 bilateral tumor-bearing mice. Vehicle, opened bars; mV01, closed bars. n = 5-6 mice/group. Data were presented as the mean ± SEM. Statistical significance was determined by an unpaired two-tailed Student's t-test.

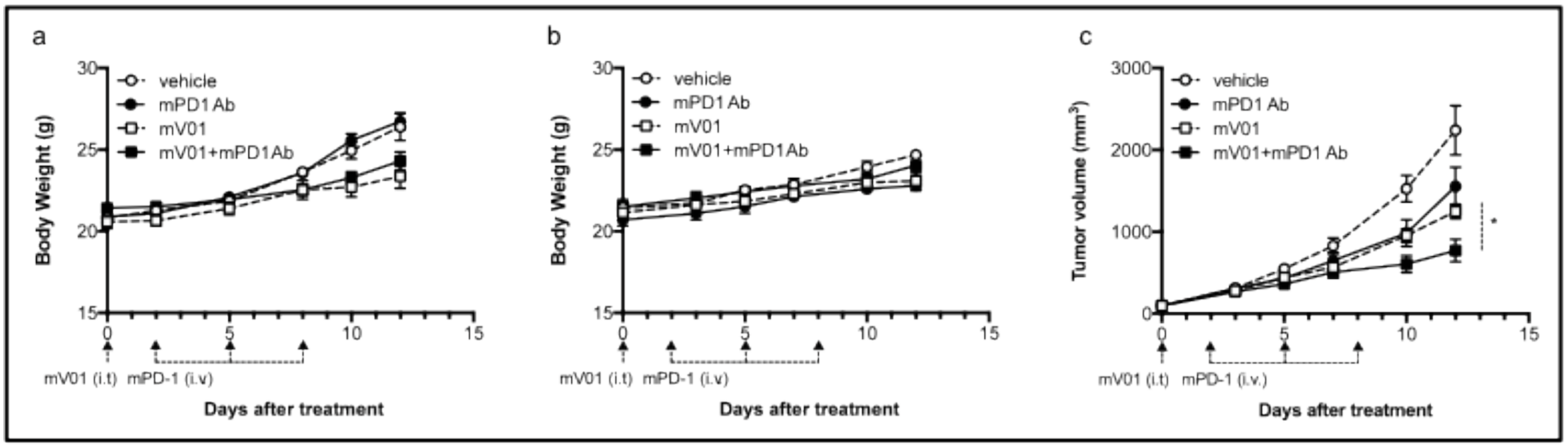

Figure S4. Effects of combination treatment on body weight and tumor progression. (a, b) Body weight of B16 tumor- (a) and MC38 tumor-bearing mice (b), and (c) tumor growth curve in MC38-bearing mice. Mice were treated with vehicle (open circles), mV01 (closed circles), anti–mPD-1 antibody (open squares), or the combination (closed squares). *n* = 8-10 mice/group. All values were presented as the mean ± SEM. **P* < 0.05 by two-way ANOVA test with Bonferroni correction.

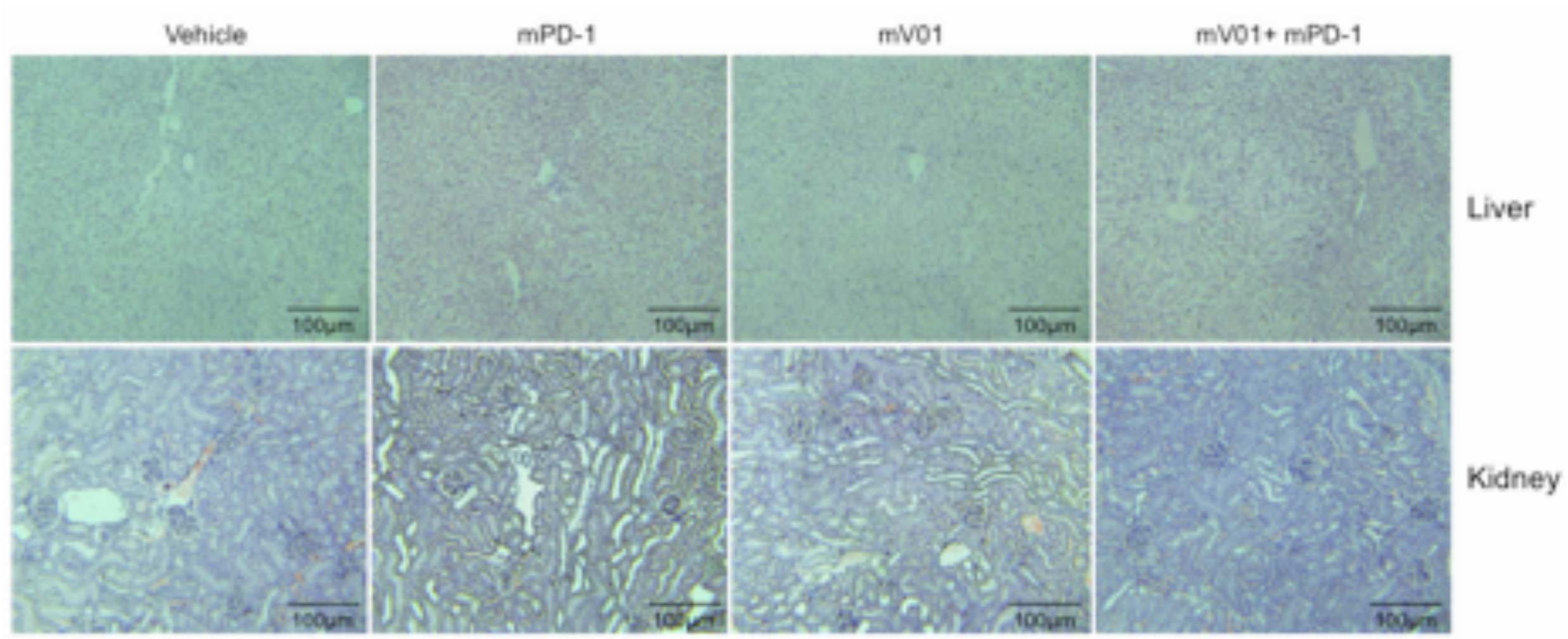

Figure S5. No toxicity effect of combination treatment in B16 tumor-bearing mice. H&E staining of Liver tissue (Top panels) and kidney tissue (bottom panels) of B16 tumor-bearing mice treated with vehicle, anti–mPD-1 antibody, mV01 and the combination. n = 8-10 mice/group. Scale bars: 100 μm.

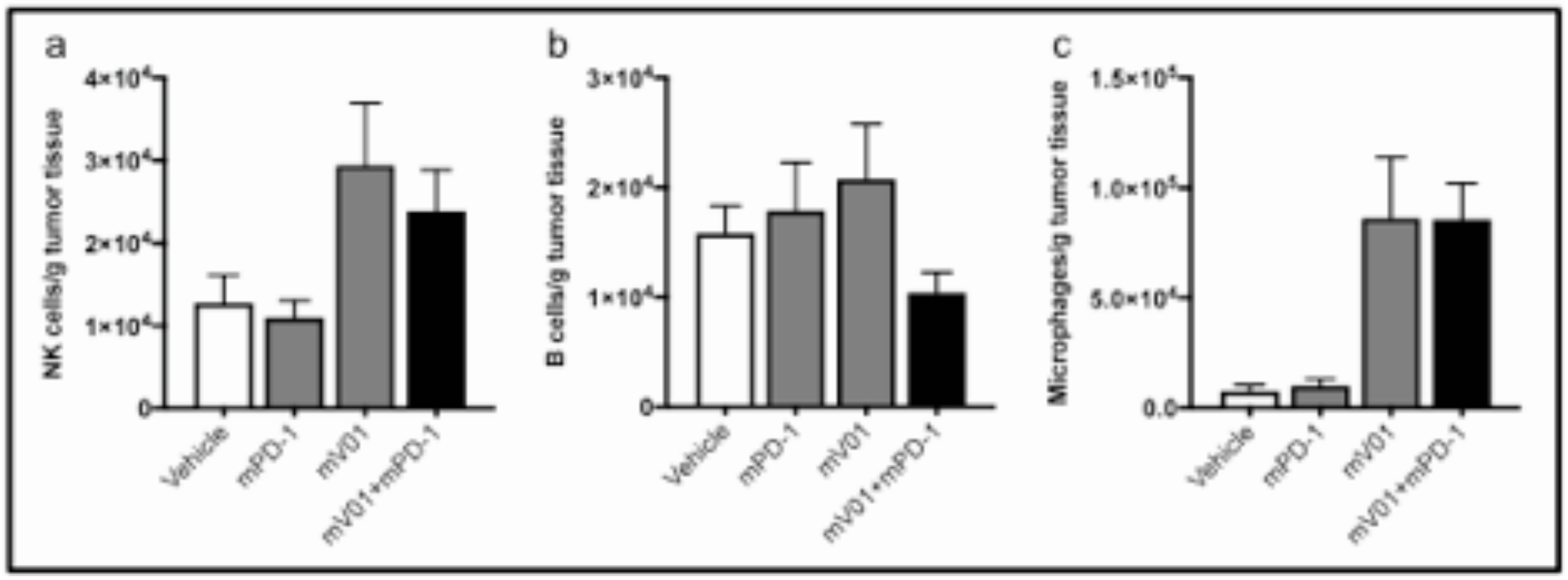

Figure S6. Quantitative analysis of (a) NK cells, (b) B cells and (c) Microphages, assessed by flow cytometry, in tumors from B16 tumor-bearing mice treated with vehicle, mV01, mPD-1 antibody and mV01 plus mPD-1 antibody. n = 10 mice/group. All values were presented as the mean ± SEM. Statistical significance was determined by one-way ANOVA.

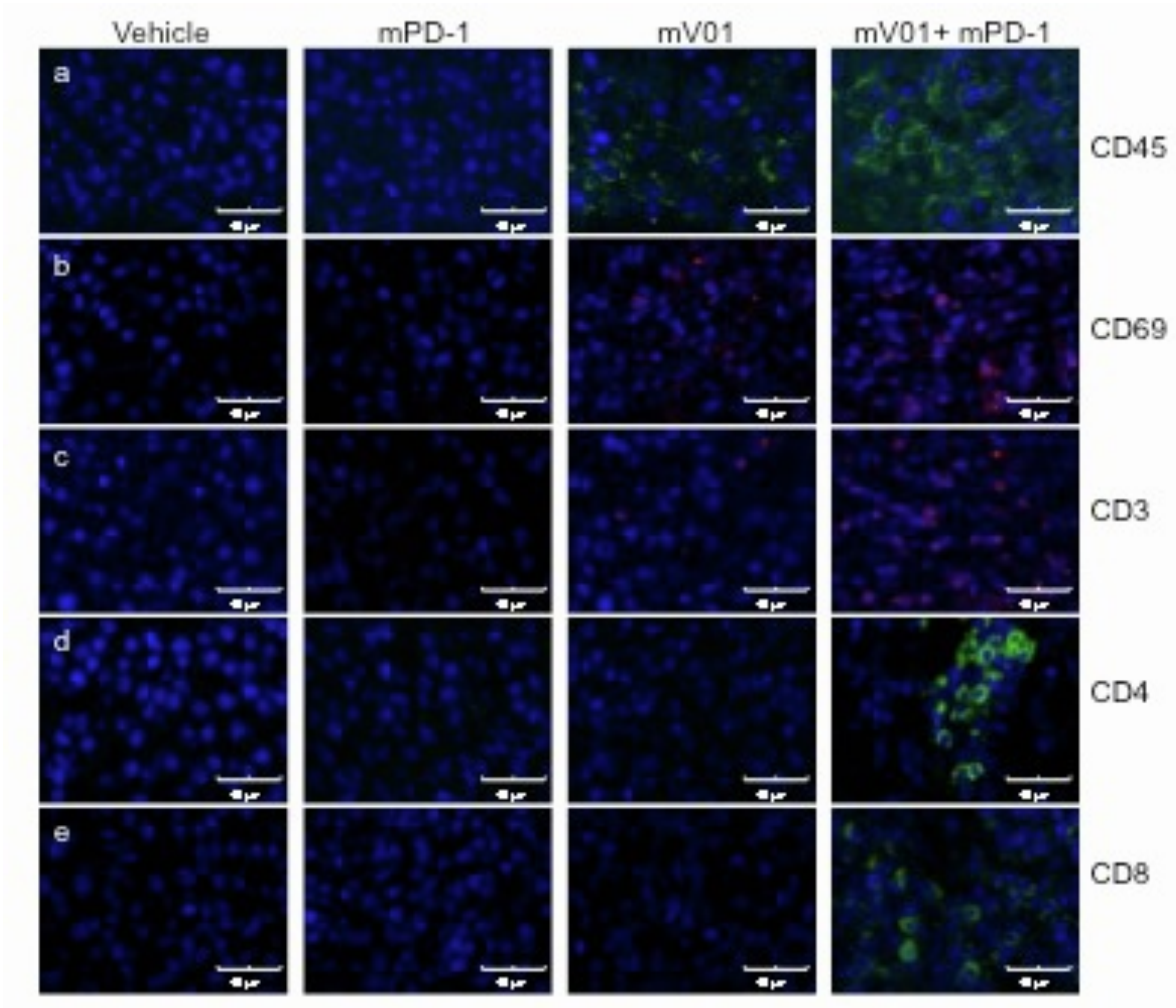

Figure S7. Blockage of PD-1 signal promoted V01-mediated anti-tumor immunity. IF staining was performed using antibodies against (a) CD45, (b) CD69, (c) CD3, (d) CD4 and (e) CD8 on the tumor sections from B16 tumor-bearing mice treated with vehicle, mPD-1 antibody, mV01, and mV01 plus mPD-1 antibody. Scale bars: 40 μm.

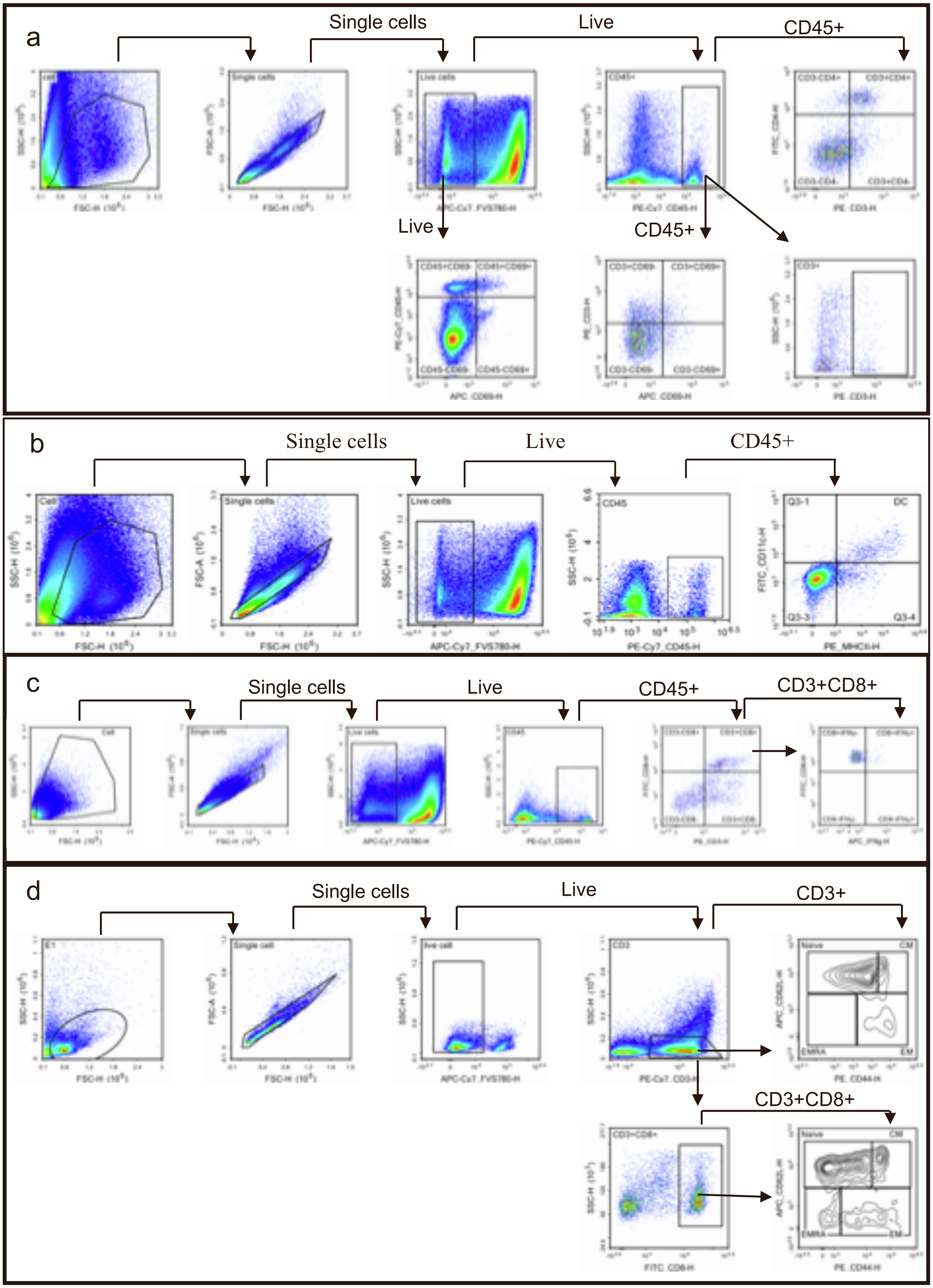
a
Single cells
Live
CD45+
Live
CD45+
b
Single cells
Live
CD45+
Cell
Single cells
Live cells
CD45
Q3-1
DC
Q3-3
Q3-4
APC-Cy7_FVS780-H
PE-Cy7_CD45-H
PE_MHCII-H
c
Single cells
Live
CD45+
CD3+CD8+
d
Single cells
Live
CD3+
CD3+CD8+

Figure S8. Gating strategy for (a) CD45+CD69+ active lymphocytes and CD45+CD3+CD69+ active T cells. In this example, CD45 PE-Cy7, CD3e PE, and CD69 APC were used. (b) CD45+CD11c+MHC II+ DC cell. In this example, CD45 PE-Cy7, CD11c FITC, and MHC II PE were used. (c) CD45+CD3e+IFN-γ+CD8a+/CD4+ IFN-γ+CD4/CD8 T cells. In this example, CD45 PE-Cy7, CD3e PE, CD8a FITC/CD4 FITC, and IFN-γ APC were used. (d) CD3+CD8+CD44-CD62L+ Naïve T cells, CD3+CD8+CD44+CD62L- TEM cells, CD3+CD8+CD44+CD62L+ TCM cells. In this example, CD3 PE-Cy7, CD8a FITC, CD44 PE, and CD62L APC were used.

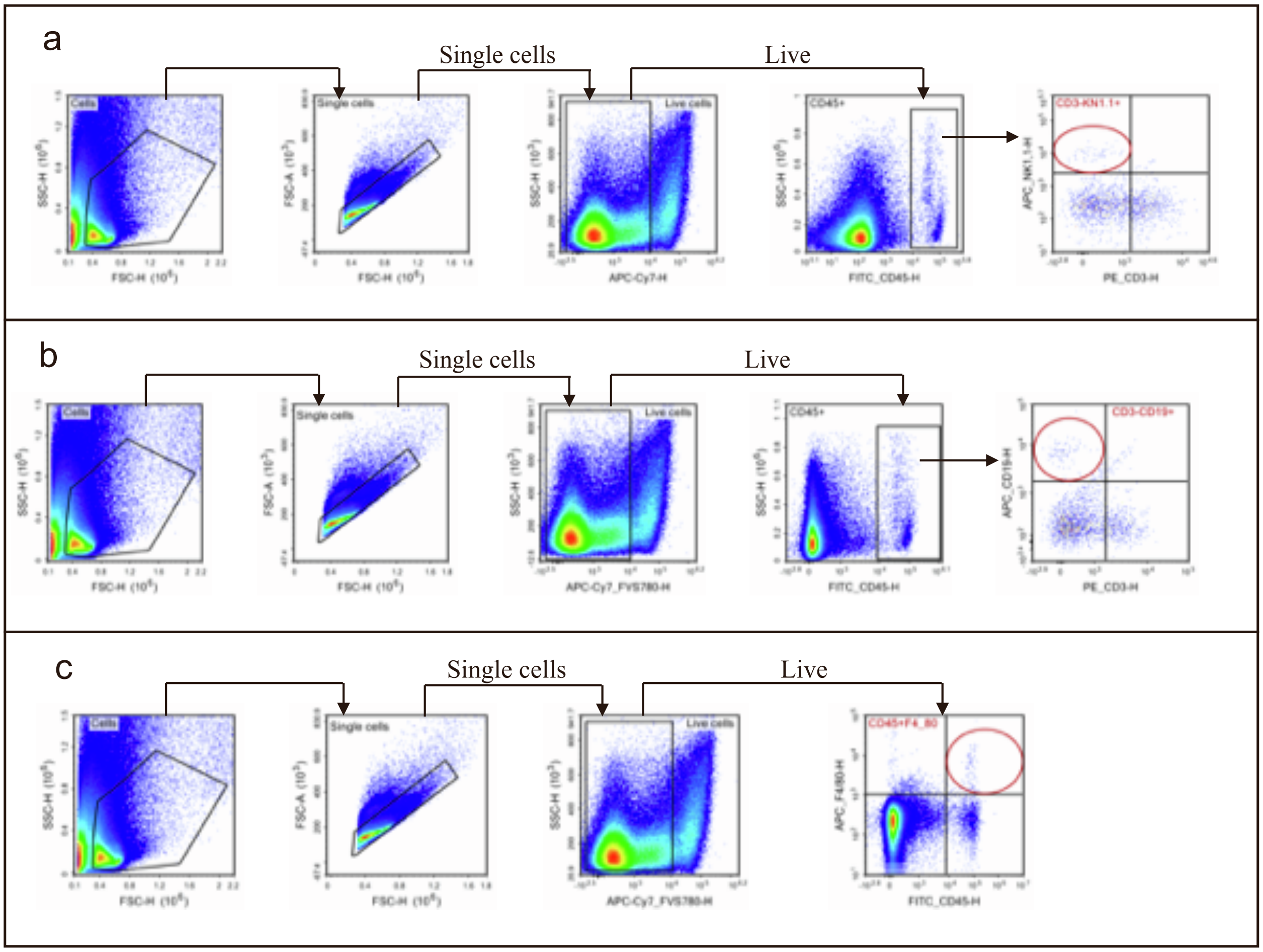

Figure S9. Gating strategy for (a) CD45+CD3-NK1.1+ NK cells. In this example, CD45 FITC, CD3e PE, and NK1.1 APC were used. (b) CD45+CD3-CD19+ B cells. In this example, CD45 FITC, CD3e PE, and CD19 APC were used. (c) CD45+F4/80+ microphages. In this example, CD45 FITC and F4/80 APC were used.

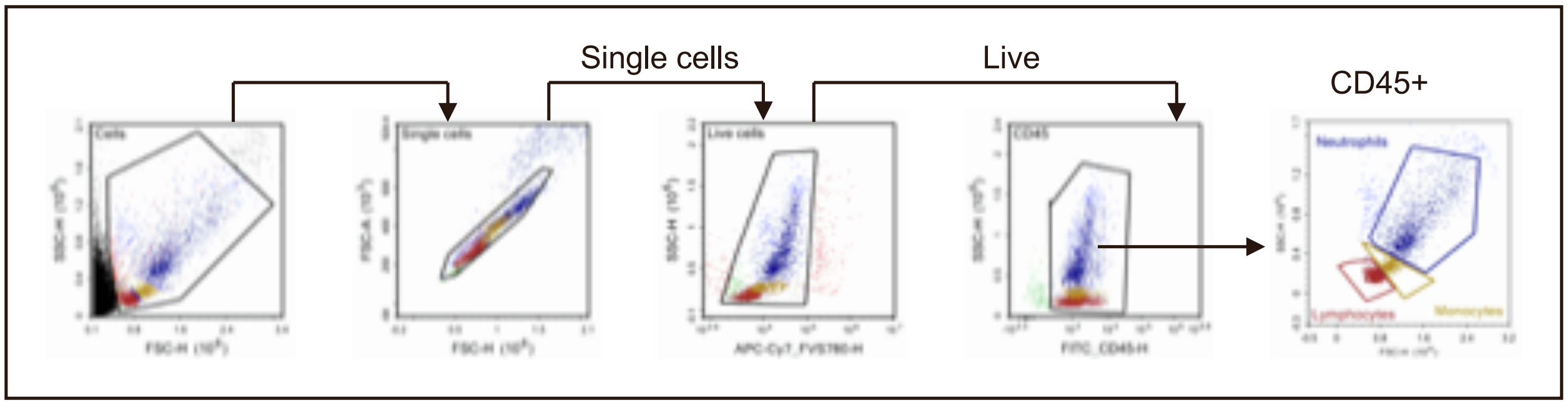

Figure S10. Gating strategy for hamster blood. The WBC fraction was incubated with FITC fluorescein-labelled anti-CD45 and subjected to a live/dead Fixable Viability Dye eFluor™ 780 staining. The major immune cell subsets, lymphocytes, monocytes and granulocytes, were gated according to their light-scattering characteristics.

Table S1 List of antibodies used in flow cytometry

Antigen	Clone	Fluorochrome	Vendor	Catalog#
CD45	30-F11	FITC	BD	553080
CD45	30-F11	PE-Cy7	eBioscience	25-0451-81
CD45	30-F11	APC	eBioscience	17-0451-82
CD11c	N418	FITC	Biolegend	117306
MHC II	NIMR-4	PE	eBioscience	12-5322-81
CD69	H1.2F3	APC	eBioscience	17-0691-82
CD3e	145-2C11	PE	eBioscience	12-0031-82
CD3	17A2	PE-Cy7	eBioscience	25-0032-82
CD4	RM4-5	FITC	eBioscience	11-0042-85
CD8a	53-6.7	FITC	Biolegend	100705
IFN-γ	XMG1.2	APC	Biolegend	505810
CD44	IM7	PE	eBioscience	12-0441-82
CD62L	MEL-14	APC	eBioscience	17-0621-82
CD86	GL1	FITC	eBioscience	25-0862-82
NK1.1	PK136	APC	BD	550627
F4/80	BM8	APC	eBioscience	17-4801-80
CD19	1D3	APC	BD	550992
CD45	HAT13A	--	Novus Biologicals	NBP2-60910
FITC Goat anti-mouse IgG (H+L)	--	FITC	Jackson	115-095-003

Table S2 Flow cytometry panel designation

Panel	Antigen	Clone	Fluorochrome
DC	CD45<br>CD11c<br>MHC II	30-F11<br>N418<br>NIMR-4	PE-Cy7<br>FITC<br>PE
CD45 activation	CD45<br>CD69	30-F11<br>H1.2F3	PE-Cy7<br>APC
T cell activation	CD45<br>CD3e<br>CD69	30-F11<br>145-2C11<br>H1.2F3	PE-Cy7<br>PE<br>APC
CD4 T cell	CD45<br>CD3e<br>CD4	30-F11<br>145-2C11<br>RM4-5	PE-Cy7<br>PE<br>FITC
CD8 T cell	CD45<br>CD3e<br>CD8a	30-F11<br>145-2C11<br>53-6.7	PE-Cy7<br>PE<br>FITC
IFNγ^+CD4 T cell	CD45<br>CD3e<br>CD4<br>IFN-γ	30-F11<br>145-2C11<br>RM4-5<br>XMG1.2	PE-Cy7<br>PE<br>FITC<br>APC
IFNγ^+CD8 T cell	CD45<br>CD3e<br>CD8a<br>IFN-γ	30-F11<br>145-2C11<br>53-6.7<br>XMG1.2	PE-Cy7<br>PE<br>FITC<br>APC
Memory T cell	CD44<br>CD62L<br>CD3<br>CD8a	IM7<br>MEL-14<br>17A2<br>53-6.7	PE<br>APC<br>PE-Cy7<br>FITC
NK Cell	CD45<br>CD3e<br>NK1.1	30-F11<br>145-2C11<br>PK136	FITC<br>PE<br>APC
Microphage	CD45<br>F4/80	30-F11<br>BM8	FITC<br>APC
B Cell	CD45<br>CD3e<br>CD19	30-F11<br>145-2C11<br>1D3	FITC<br>PE<br>APC
